# The role of DDK and Treslin–MTBP in coordinating replication licensing and pre-initiation complex formation

**DOI:** 10.1098/rsob.210121

**Published:** 2021-10-27

**Authors:** Ilaria Volpi, Peter J. Gillespie, Gaganmeet Singh Chadha, J. Julian Blow

**Affiliations:** Centre for Gene Regulation and Expression, School of Life Sciences, University of Dundee, Dundee DD1 5EH, UK

**Keywords:** MTBP, Treslin, TopBP1, DDK, PP1, simurosertib

## Abstract

Treslin/Ticrr is required for the initiation of DNA replication and binds to MTBP (Mdm2 Binding Protein). Here, we show that in *Xenopus* egg extract, MTBP forms an elongated tetramer with Treslin containing two molecules of each protein. Immunodepletion and add-back experiments show that Treslin–MTBP is rate limiting for replication initiation. It is recruited onto chromatin before S phase starts and recruitment continues during S phase. We show that DDK activity both increases and strengthens the interaction of Treslin–MTBP with licensed chromatin. We also show that DDK activity cooperates with CDK activity to drive the interaction of Treslin–MTBP with TopBP1 which is a regulated crucial step in pre-initiation complex formation. These results suggest how DDK works together with CDKs to regulate Treslin–MTBP and plays a crucial in selecting which origins will undergo initiation.

## Graphical abstract

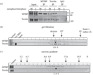

## 1. Introduction

The initiation of eukaryotic DNA replication occurs at replication origins that have been licensed by being loaded with double hexamers of MCM2–7 proteins. The combined action of two kinases, DDK and S-CDK, then triggers the recruitment of Cdc45 and the GINS complex onto these MCM2-7 double hexamers to form the replicative CMG (Cdc45–MCM–GINS) helicase. *In vitro* experiments in *Xenopus* egg extract have shown that DDK activity is required before S-CDK activity [1,2]. DDK-mediated hyperphosphorylation of Mcm4 but not Mcm2 correlates with replication initiation in both *Xenopus* egg extract and human cells [3,4]. In *Saccharomyces cerevisiae*, DDK phosphorylation of MCM2–7 promotes the recruitment of Sld3 and Cdc45 to origins, and then S-CDK phosphorylation of Sld3 and Sld2 allows the recruitment of Dbp11, Sld2, Sld3, GINS and Pol ε [5–8]. DDK-dependent MCM phosphorylation is reversed by protein phosphatase 1 targeted to chromatin by Rif1 in *Xenopus* egg extract and in both *S. cerevisiae* and human cells [3,4,9,10].

Sld7 was identified in *S. cerevisiae* as a gene that is synthetically lethal with Dpb11 [11,12]. Despite Sld7 not being absolutely required for DNA replication, it physically interacts with Sld3 and is required for the proper function of Sld3 at initiation [8,12,13]. Sld7 mutants have reduced total levels of Sld3 and delayed dissociation of Sld3 from GINS at replication initiation [12]. The crystal structures obtained from Sld3 and Sld7 domains suggest a model of interaction between these proteins in which two molecules of Sld7 interact with each other in an antiparallel manner through their C-terminal domains [14]. The N-terminal domain of Sld7 interacts with the N-terminal domain of Sld3 to form a tetramer in which the two molecules of Sld3 are connected by the two molecules of Sld7 [14]. This conformation could allow the correct positioning of Sld3 onto the double hexamer of MCM2–7 in order to promote bidirectional firing of the origins.

MTBP was identified in a yeast two-hybrid screen as an interacting partner of MDM2, the E3 ubiquitin ligase that targets p53 for destruction [15]. In human cells, knockdown of MTBP blocks cell growth and induces a G1 arrest [15,16]. It is frequently found overexpressed in cancer [17,18]. In human cells, MTBP has been identified as an interacting partner of Treslin, the metazoan homologue of Sld3, throughout the cell cycle [19]. An MTBP-interacting domain has been identified on Treslin, upstream of the Cdc45-interacting domain [19]. Treslin lacking the MTBP-interacting domain is unable to rescue the DNA replication phenotype of Treslin-depleted cells, indicating that the Treslin–MTBP interaction is essential for replication [19]. MTBP siRNA causes the impairment of CMG assembly and the lengthening of S phase, suggesting a direct role in the initiation of DNA replication [19].

Treslin interacts with TopBP1, the metazoan homologue of Dpb11. CDK-dependent phosphorylation of the well-conserved S1001 of Treslin mediates its interaction with the phospho-binding BRCT domains of TopBP1 [19,20]. Mutation of this residue, but not of the adjacent well-conserved CDK consensus-site T969, significantly reduced the interaction of Treslin with TopBP1 and cells harbouring a non-phosphorylatable mutation of S1001 were deficient in DNA replication [20]. The formation of a tripartite Treslin–MTBP–TopBP1 complex in human cells is curtailed when Treslin's conserved phosphosites are mutated [19]. CDK phosphorylation of Treslin is therefore required both for the interaction of Treslin–MTBP with TopBP1 and to support DNA replication in human cells. Consistent with this, cells with a phosphomimetic Treslin mutant display faster replication kinetics and a shorter S phase [21]. In addition to Treslin–MTBP, RecQ4, the higher eukaryotic homologue of yeast Sld2, is a potential CDK substrate. Following phosphorylation of RecQ4, it associates with licensed chromatin, together with Treslin–MTBP, to form the pre-initiation complex (pre-IC) [22].

Here, we further characterize the role of MTBP in DNA replication using the *Xenopus* egg extract cell-free system. Our work complements and extends a recent report describing *Xenopus* MTBP [23]. We show that MTBP and Treslin form a tetrameric complex that is required for the initiation of DNA replication, similar to Sld3/Sld7 in yeast. However, in this system, the regulation of MTBP binding onto chromatin appears substantially different from Sld7 in yeast. We find that DDK activity is required to both increase and strengthen the interaction of Treslin–MTBP with chromatin and to mediate its association with TopBP1. Our data suggest a key role for DDK activity in coordinating the interaction of Treslin–MTBP with licensed replication origins and TopBP1, thereby determining which origins are selected to undergo replication initiation.

## 2. Results

### 2.1. Characterization of MTBP and Treslin antibodies

In human cells, MTBP has been identified as an interacting partner of Treslin involved in the regulation of the initiation of DNA replication [19]. To identify how MTBP is involved in DNA replication, we studied its role and regulation in *Xenopus* egg extracts which support complete genome duplication *in vitro* [24]. We raised antibodies against the C′ and N′ termini of *Xenopus* MTBP, which we denote as MTBP-1 and MTBP-2, respectively. We also used a commercial antibody raised against a conserved central region of the human protein, which we denote by MTBP-S. The antibodies were used to immunoblot whole *Xenopus* egg extract, replicating chromatin and immunoprecipitates (electronic supplementary material, figure S1a). While all the antibodies detected multiple bands in whole extract, they each recognized the same single band on replicating chromatin of the expected molecular weight for MTBP. Importantly, they each also specifically immunoprecipitated the same band. We also raised two antibodies against *Xenopus* Treslin (Treslin-1 and Treslin-2), which showed a similar specificity (electronic supplementary material, figure S1b), and an antibody against *Xenopus* TopBP1, which recognized only a single band of the expected molecular weight in both whole egg extract and on isolated chromatin (electronic supplementary material, figure S1c). To confirm the identity of the major band identified by the MTBP and Treslin antibodies, we performed mass spectrometry of the immunoprecipitates, which confirmed that MTBP and Treslin were the major components of these samples (electronic supplementary material, figure S2).

### 2.2. A Treslin–MTBP complex in *Xenopus* egg extract

**Figure 1.**
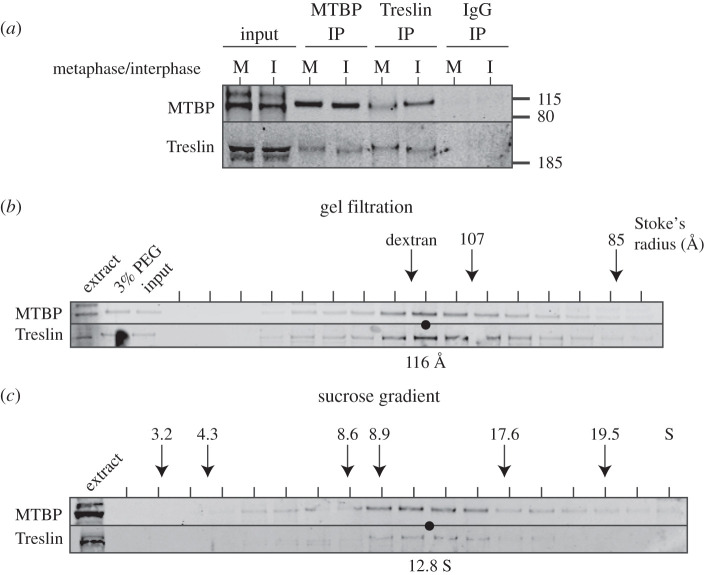
Treslin–MTBP complex in *Xenopus* egg extract. (*a*) Immunoprecipitation of MTBP and Treslin from metaphase (M) or interphase (I) extract using affinity purified MTBP-1 and Treslin-1 antibodies, immunoblotted for MTBP and Treslin. (*b*) Gel filtration of 3% PEG-precipitated proteins from *Xenopus* egg extract immunoblotted for MTBP and Treslin. The position and calculated Stoke's radius of the Treslin–MTBP peak are indicated. (*c*) 5–40% sucrose gradient centrifugation of 3% PEG-precipitated proteins, immunoblotted for MTBP and Treslin. The position and calculated sedimentation coefficient of the Treslin–MTBP peak is indicated.

To identify a possible Treslin–MTBP complex, we immunoprecipitated Treslin and MTBP from aliquots of *Xenopus* egg extract in either metaphase or interphase (figure 1*a*). Immunoblotting showed that MTBP antibodies co-precipitated Treslin and Treslin antibodies co-precipitated MTBP. These results indicate that in *Xenopus* egg extract, MTBP and Treslin form a complex throughout the cell cycle. This is consistent with previous reports in *Xenopus* egg extracts [23] and human cells [19], and resembles the interaction between Sld3 and Sld7 in yeast [12,13].

**Figure 2.**
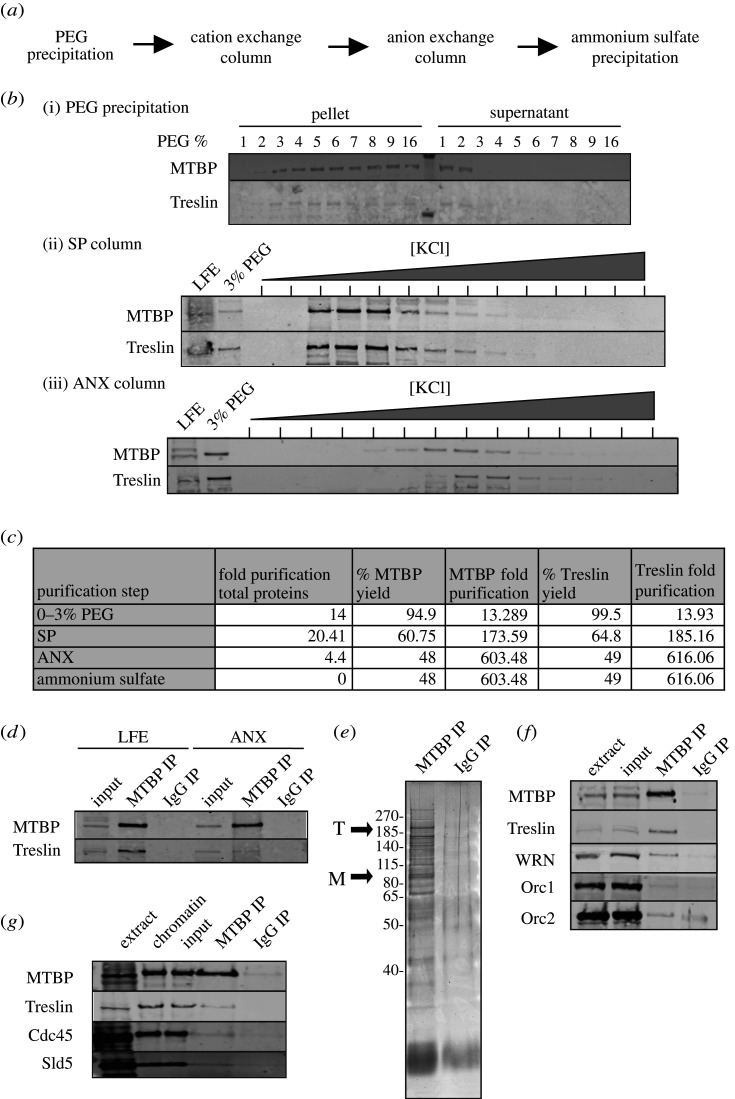
Treslin–MTBP complex purification. (*a*) Flow chart of the purification process. (*b*) Immunoblots of MTBP and Treslin present in (i) PEG, (ii) SP and (iii) ANX fractions. (*c*) Table of purification achieved for MTBP and Treslin. (*d,e*) Purified material was immunoprecipitated using MTBP-1 affinity purified antibody. Samples were immunoblotted for MTBP and Treslin (*d*) and run on a NuPage gel and stained with SYPRO Ruby (*e*). (*f*) MTBP immunoprecipitate from whole extract was run on NuPage gels and immunoblotted for the indicated proteins. (*g*) Sperm nuclei were incubated in interphase extract for 30 min; chromatin was isolated, digested with DNAse and immunoprecipitated with MTBP-1 or non-immune antibodies. Immunoprecipitates were immunoblotted for the indicated proteins.

We next set out to determine the molecular weight of the Treslin–MTBP complex. Elongated proteins appear larger than spherical proteins of the same molecular weight when analysed by size exclusion chromatography, but appear smaller when analysed by sucrose gradient centrifugation. However, by combining data from both separation techniques (Stoke's radius and sedimentation coefficient) and using the Siegel & Monty equation [25], the native molecular weight of a protein or protein complex can be determined in a manner that is unaffected by its shape. We precipitated proteins from a clarified supernatant of whole egg extract using polyethylene glycol (PEG) (figure 2), selected the protein fraction containing MTBP and Treslin, then analysed it by size exclusion chromatography and sucrose gradient centrifugation. MTBP and Treslin showed similar elution profiles in size exclusion chromatography and sucrose gradient centrifugation, suggesting that in egg extract, they form a single multimeric species. This complex has a Stoke's radius of 116 Å (figure 1*b*) and a sedimentation coefficient of 12.8 S (figure 1*c*). Applying the Siegel and Monty equation gives a molecular weight for the Treslin–MTBP complex of 626 kDa. This is close to (± less than 1%) the expected molecular weight of a 632 kDa tetramer composed of two molecules of MTBP (96 kDa) and two molecules of Treslin (220 kDa). The Treslin–MTBP complex appears larger on gel filtration than thyroglobulin tetramer (1338 kDa, 107 Å) and smaller on sucrose gradients than β-amylase (200 kDa, 54 Å, 8.9 S), suggesting that Treslin–MTBP is highly elongated.

Quantitative immunoblotting that compares the signal of MTBP in extract with the signal from known quantities of the MTBP-2-antigen suggested that the concentration of MTBP in undiluted egg extract is approximately 0.2 µg ml^−1^ or approximately 2 nM (electronic supplementary material, figure S3). The concentration of the proposed Treslin–MTBP tetramer is, therefore, approximately 1 nM. *Xenopus* egg extract can efficiently replicate approximately 30 ng DNA µl^−1^ of extract [26] with active replication origins spaced approximately 10 kb apart [27]. At 30 ng DNA µl^−1^, the concentration of active origins is, therefore, approximately 4 nM, suggesting that Treslin–MTBP is involved in replication initiation at rate-limiting levels.

### 2.3. Purification of Treslin–MTBP complex

To investigate the composition of the Treslin–MTBP complex, we carried out a partial chromatographic purification (figure 2*a*). First, we precipitated proteins from a clarified supernatant of whole egg extract using PEG (figure 2*b*(i)), then used cation and anion exchange chromatography to further enrich MTBP and Treslin (figure 2*b*(ii,iii)). We carried out a final ammonium sulfate precipitation in order to concentrate the protein fractions. During the purification, we achieved a 1200-fold reduction of total protein as estimated by UV absorbance at 280 nm with a yield of MTBP and Treslin of 48% and 49%, respectively (figure 2*c*). Combining data for the yield with protein concentration measurements suggests that MTBP and Treslin were both enriched greater than 600-fold (figure 2*c*).

Immunoprecipitation of the enriched material with MTBP-1 antibodies co-precipitated Treslin, though less efficiently than in whole extract, suggesting that the complex was only partially maintained throughout the chromatographic procedures (figure 2*d*); consistent with this a fraction of MTBP separate from Treslin is found on cation exchange chromatography (figure 2*b*(iii)). Total protein staining of the immunoprecipitates showed major bands at the expected position of MTBP and Treslin and as expected, the higher molecular weight Treslin band is the more intense (figure 2*e*). Mass spectrometry confirmed that MTBP and Treslin were the major protein components of the immunoprecipitate (electronic supplementary material, figure S4). We were unsuccessful in reducing the number of bands in the immunoprecipitates by increasing salt concentration during the wash steps, because this strongly reduced the co-precipitation of Treslin with MTBP antibodies. Taken together, our results strongly suggest that MTBP and Treslin form a 2 : 2 heterodimer, though the partial purification falls short of providing definitive evidence for this.

Mass spectrometry of the MTBP immunoprecipitate identified other proteins, including components of the origin recognition complex (ORC), Werner's helicase (WRN) and Topoisomerase IIα. To test whether any of these interactions were specific, we immunoblotted MTBP-1 immunoprecipitates with antibodies to these proteins. Orc1, Orc2 and WRN were detected in MTBP-1 immunoprecipitates (figure 2*f*). We therefore conclude that although MTBP and Treslin exist primarily as a hetero-tetramer in egg extract, they can also associate with other proteins to a limited extent.

The Treslin–MTBP hetero-tetramer is present in interphase egg extracts (figure 1). Following nuclear formation, these extracts support one complete round of DNA replication. To determine whether the Treslin–MTBP complex is present on chromatin during S phase, we performed a chromatin immunoprecipitation (ChIP) from replicating nuclei isolated from egg extract (figure 2*g*). We digested replicating chromatin with nuclease and performed MTBP immunoprecipitation. Treslin was also co-precipitated with MTBP, at levels comparable to the total amount loaded onto chromatin, suggesting that the two proteins remain associated with one another when bound to DNA. This also suggests that, consistent with the chromatography data, there is no significant excess of Treslin over MTBP in the extract. MTBP also co-precipitated Cdc45 and Sld5 on chromatin (figure 2*g*); these proteins are members of the CMG helicase, consistent with the idea that the Treslin–MTBP complex is involved in assembling the CMG complex.

### 2.4. Regulation of MTBP recruitment onto chromatin

**Figure 3.**
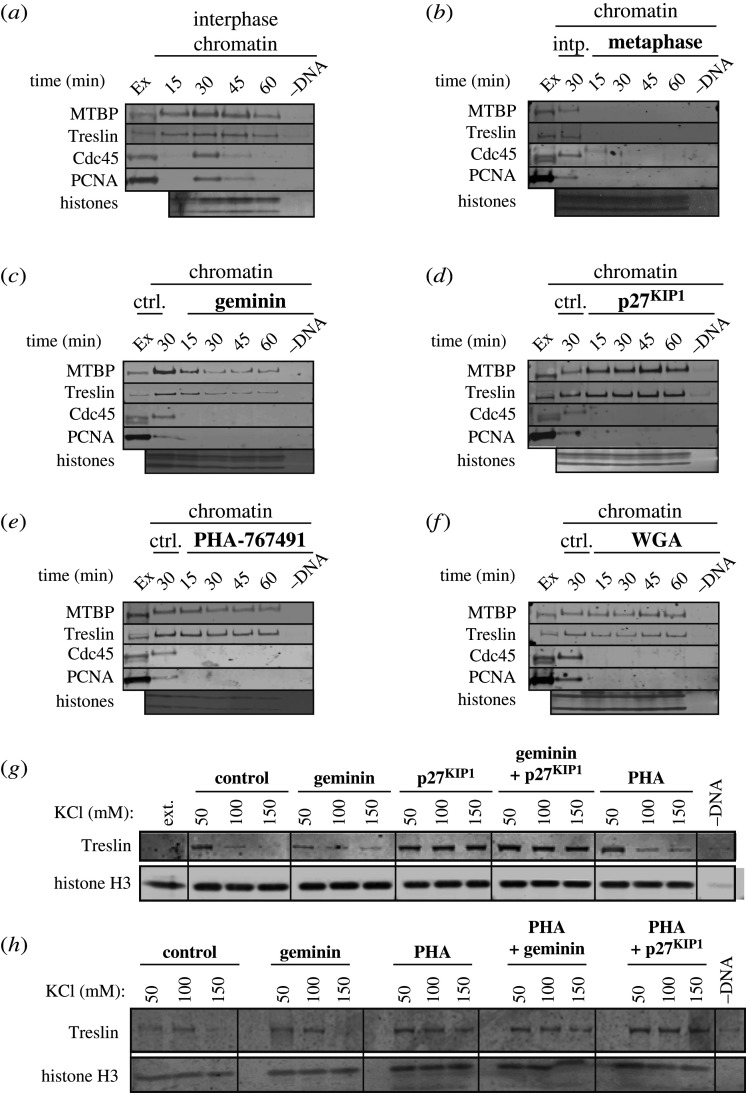
MTBP loading onto chromatin in metaphase and interphase extract. (*a,b*) Sperm nuclei were incubated in interphase (*a*) or metaphase (*b*) extract for the indicated times; chromatin was isolated, run on NuPage gels and immunoblotted for the indicated proteins. The lower portion of the gel was stained with SYPRO Ruby to detect histones. Ex, extract control. (*c–f*) Sperm nuclei were incubated in interphase extract for the indicated times in the presence of either: (*c*) geminin, (*d*) p27^KIP1^, (*e*) PHA-767491 or (*f*) wheat germ agglutinin (WGA); chromatin was isolated, run on NuPage gels and immunoblotted for the indicated proteins. The lower portion of the gel was stained with SYPRO Ruby to detect histones. Ex, extract control; ctrl, control chromatin incubated for 30 min in interphase extract without drug treatment. (*g*,*h*) Sperm nuclei were incubated in interphase extract for 60 min in the presence of either geminin, p27^KIP1^, PHA or a combination of two of these; chromatin was isolated at the indicated salt concentration, run on a NuPage SDS–PAGE gel and immunoblotted for Treslin. The lower portion of the gel was immunoblotted for Histone H3 to serve as a chromatin loading control.

As a first step to characterising the function of MTBP in DNA replication, we analysed the chromatin recruitment of MTBP and Treslin at various times after adding sperm nuclei to interphase extract. MTBP and Treslin were recruited onto chromatin at 15 min, during the licensing phase and before replication initiation, when Cdc45 and PCNA were not yet present on chromatin (figure 3*a*). The MTBP and Treslin signal peaked at 30 min during S phase, when CMG proteins (Cdc45, Psf2 and Sld5), pre-IC proteins (TopBP1, RecQ4, Mcm10 and AND1/Ctf4) and DNA synthesis proteins (RPA, PCNA and the three replicative polymerases α, β and ε) were present on chromatin, and decreased during the later stages of S phase consistent with these (figure 3*a*; electronic supplementary material, figure S5). By contrast, when sperm chromatin was incubated in metaphase-arrested extract, neither MTBP nor Treslin were recruited to the chromatin (figure 3*b*). These results indicate that MTBP and Treslin bind chromatin before S phase starts and peak during S phase, consistent with them being involved in replication initiation.

We next carried out chromatin isolation from interphase extract supplemented with geminin to block replication licensing and prevent the MCM2–7 complex from loading onto chromatin [28–30]. Figure 3*c* and electronic supplementary material, figure S6a show that whereas geminin significantly inhibited MTBP and Treslin recruitment onto chromatin, which peaked at 15 min and then declined, all CMG helicase and all other pre-IC proteins are unable to associate with chromatin when licensing is inhibited. A similar decrease in MTBP and Treslin association with chromatin was observed in extract treated with RL5a, a small molecule which inhibits the ability of ORC to bind productively with DNA and which thereby inhibits origin licensing [31] (electronic supplementary material, figure S6).

To verify whether MTBP requires S phase kinase activity to load onto chromatin, we inhibited the DDK and CDK activities that are required for the initiation of DNA replication in *Xenopus* egg extract [1,2,32–34]. Treslin is a substrate for S phase CDKs, and its phosphorylation is required for it to bind to Dpb11/Cut5/TopBP1 at replication origins [20,35–39]. DDK is recruited to MCM2–7 at licensed origins where it phosphorylates Mcm2 and Mcm4 [1–4].

Figure 3*d* and electronic supplementary material, figure S6b show that in extract treated with the CDK inhibitor p27^KIP1^, the Cdc45 and GINS (Psf2 and Sld5 proteins) components of CMG, pre-IC protein TopBP1 and the proteins required for DNA synthesis do not associate with chromatin, but MTBP and Treslin were loaded onto chromatin to higher levels compared to the control, consistent with a previous report that inhibition of CDKs causes a hyper-loading of Treslin in *Xenopus* egg extracts [38]. This indicates that neither MTBP nor Treslin need S phase CDK activity to load onto chromatin, and that CDK activity actually reduces their loading. RecQ4 was also loaded onto chromatin at near normal levels when CDK activity was inhibited (electronic supplementary material, figure S6).

When PHA-767491 was added to *Xenopus* extract to inhibit DDK activity [3,4,40], MTBP and Treslin peaked on chromatin at 15 min at levels comparable to the control, but then declined, whereas other than the MCM proteins, all other components of CMG, all other pre-IC and the proteins required for DNA synthesis did not associate (figure 3*e*; electronic supplementary material, figure S6c). When we used WGA to inhibit nuclear pore function [41]—thereby reducing the effective concentration of replication factors around DNA—Treslin–MTBP recruitment stayed at an approximately constant level comparable to the peak seen in control extract (figure 3*f*). This could be explained by the opposing effects of reducing the concentrations of Treslin–MTBP and DDK concentrations around DNA which would tend to reduce Treslin–MTBP loading, while at the same time reducing CDK activity around DNA which would tend to increase Treslin–MTBP loading.

The reduced chromatin association of Treslin–MTBP in extracts treated with either geminin or RL5a to inhibit licensing and PHA-767491 to inhibit DDK activity is in stark contrast to the hyperloading seen when CDK activity is inhibited by p27^KIP1^ (figure 3; electronic supplementary material, figures S5 and S6). This removal of Treslin–MTBP from chromatin following the peak association at 15 min and hyperloading is consistent with nuclear formation and the concomitant increase of local CDK activity. To determine the combined effect of inhibiting both licensing and either CDK or DDK activity, we isolated chromatin from egg extract, in increasing concentrations of salt, that had been treated either with p27^KIP1^, geminin or PHA-767491 alone or in combination; chromatin isolated in increasing concentrations of KCl allows discrimination between different states of protein chromatin association [42]. Figure 3*g* shows that irrespective of whether replication licensing has occurred, inhibition of CDK both increases and stabilizes the association of Treslin–MTBP with chromatin. Figure 3*h* shows that the initial binding of Treslin to chromatin is largely unaffected by licensing or DDK inhibition, but is still stimulated by CDK inhibition when DDK is inhibited. Taken together, these results suggest that CDKs play a role in destabilizing the interaction between Treslin–MTBP and chromatin that is largely independent of other replication activities.

### 2.5. Involvement of MTBP in DNA replication

**Figure 4.**
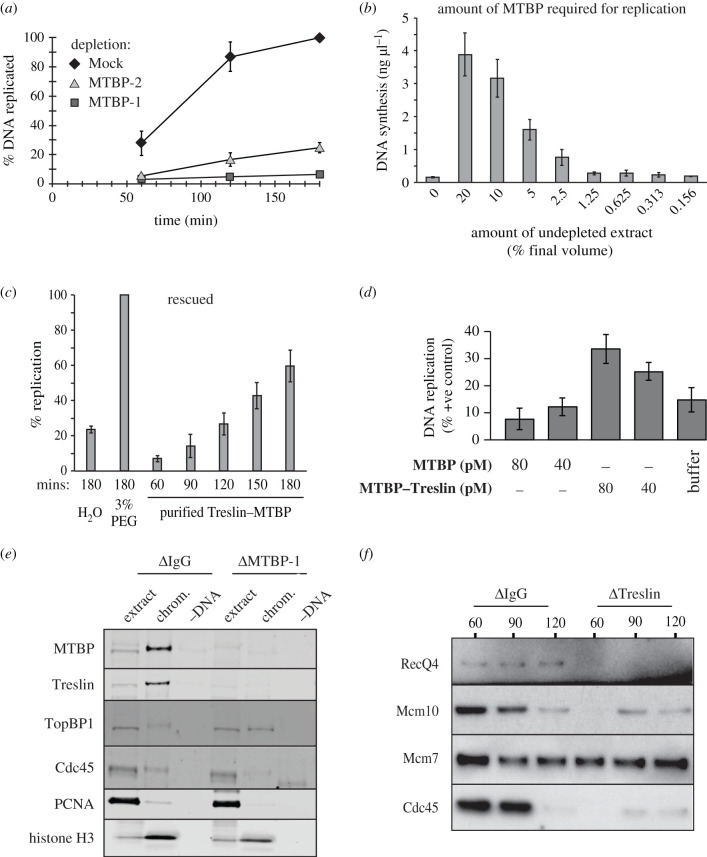
Immunodepletion of MTBP from *Xenopus* egg extract. (*a*) DNA replication was assayed in the indicated depleted extracts (average of three independent experiments, error bars represent standard error). (*b*) Undepleted extract was titrated into MTBP-1-depleted extract and DNA synthesis was assayed after 3 h. The average of three independent experiments; error bars represent standard error. Sperm nuclei were added to a final concentration of 3 ng µl^−1^. (*c*) MTBP-1-depleted extract was incubated with H_2_O, 3% PEG or ammonium sulfate concentrated partially purified MTBP–Treslin complex diluted in H_2_O (300 pM). Replication was assayed at the indicated times. Average of three independent experiments; error bars represent standard error. (*d*) MTBP or Treslin–MTBP was delivered to MTBP-1-depleted extract at either 40 or 80 pM. Sperm chromatin was added and replication was assayed after 3 h, expressed as a percentage of the replication achieved in depleted extract supplemented with an excess of the 3% PEG fraction. Average of three independent experiments; error bars represent standard error. (*e*) Mock or MTBP-1-depleted extract were supplemented plus or minus sperm chromatin; after 90 min chromatin was isolated, run on a NuPage gel and immunoblotted for the indicated proteins. (*f*) Mock or Treslin-1-depleted extract were supplemented with sperm chromatin; chromatin was isolated at the indicated times, run on a NuPage gel and immunoblotted for the indicated proteins.

To verify the involvement of MTBP in DNA replication, we immunodepleted MTBP from *Xenopus* egg extract using the MTBP-1 and MTBP-2 antibodies. The residual MTBP and Treslin in the depleted extracts was approximately 5% (electronic supplementary material, figure S7a). Extracts depleted with MTBP-1 or MTBP-2 antibodies showed a strongly reduced ability to support DNA replication (figure 4*a*). To verify that both the MTBP-1 and MTBP-2 antibodies depleted the same factor required for replication, we performed complementation assays, where equal volumes of depleted extract were mixed and then subjected to a replication assay. Mixtures of mock-depleted extract and MTBP-1 or MTBP-2 depleted extracts supported replication, while mixtures of MTBP-1 and MTBP-2 depleted extracts did not (electronic supplementary material, figure S7b). This indicates that the MTBP-1 and MTBP-2 antibodies deplete the same essential factors required for DNA replication.

Even though in MTBP-depleted extracts, DNA replication was significantly reduced the depleted extracts could still efficiently support complementary strand synthesis on single-stranded M13 plasmid DNA (electronic supplementary material, figure S7c) and nuclear assembly was not impaired (electronic supplementary material, figure S7d). To determine the amount of Treslin–MTBP complex required for DNA replication, we performed serial dilutions of normal extract into MTBP-depleted extracts and tested the amount of DNA synthesis (figure 4*b*). This showed that 5% by volume of undepleted extract could rescue replication to an efficiency of approximately 50% in the depleted extracts. This is equal to an MTBP concentration of approximately 0.1 nM and a Treslin–MTBP tetramer concentration of approximately 0.05 nM. Given the significantly longer S phase observed in immunodepleted extracts, this is consistent with the hypothesis that MTBP is present at limiting levels for replication initiation.

We next carried out a rescue experiment adding an aliquot of partially purified Treslin–MTBP (figure 2) to extract depleted with MTBP antibodies. Ammonium sulfate precipitation was used to concentrate the Treslin–MTBP sufficiently to allow assay, but residual ammonium sulfate in the sample was somewhat inhibitory to DNA replication. As positive control for rescue, we used the maximum rescue achieved by Treslin–MTBP concentrated after precipitation with PEG (directly from extract; figure 4*c*). The fraction containing the 600-fold enriched Treslin–MTBP complex rescued replication to 60% compared to the positive control. This highly efficient rescue strongly suggests that Treslin–MTBP is the replication factor missing in the depleted extracts.

We next asked whether MTBP alone is sufficient to rescue the replication phenotype of the MTBP-depleted extract, or whether the Treslin–MTBP complex is required. During anion exchange chromatography, approximately 20% MTBP elutes before the Treslin peak, suggesting that MTBP is in slight excess in egg extract (figure 2). We compared the ability of fractions containing MTBP only or the Treslin–MTBP complex (figure 4*d*; electronic supplementary material, figure S7e) to rescue DNA replication in MTBP-depleted extract. While fractions containing Treslin–MTBP could rescue DNA replication to approximately 30% of the positive control, comparable amounts of MTBP in the MTBP-only fraction were not able to rescue replication at all. These results suggest that MTBP alone is not sufficient to rescue the replication phenotype of the MTBP-depleted extract and that Treslin is an essential factor for replication.

**Figure 5.**
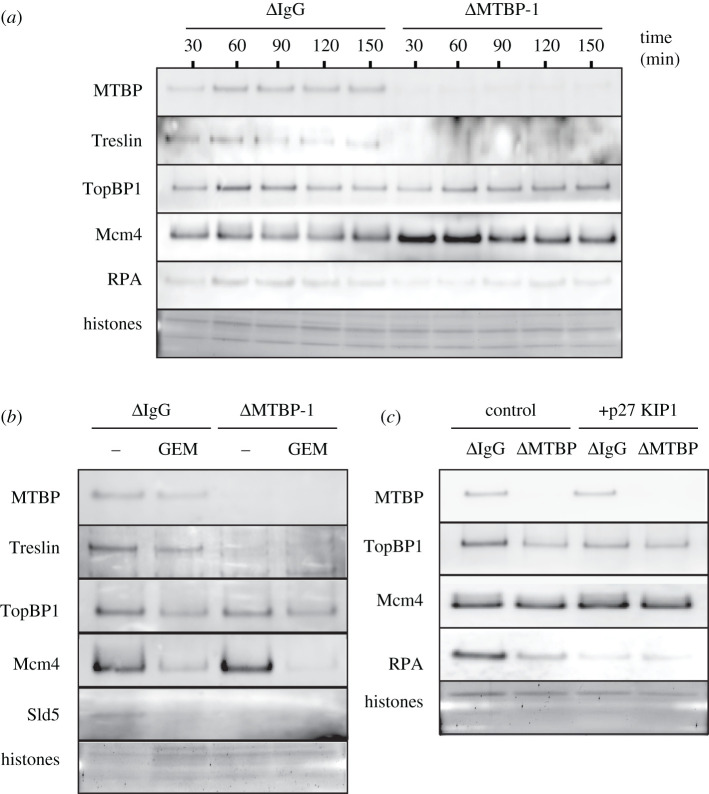
TopBP1 chromatin association in the absence of MTBP. (*a*) Mock or MTBP-1-depleted extract was incubated with sperm chromatin for the indicated times, chromatin was isolated, run on a NuPage gel and immunoblotted for the indicated proteins. The lower portion of the gel stained with SYPRO Ruby to detect histones. (*b*,*c*) Mock or MTBP-1-depleted extract were supplemented with sperm chromatin and either ± (*b*) geminin or (*c*) p27^KIP1^; after 60 min chromatin was isolated, run on a NuPage gel and immunoblotted for the indicated proteins. The lower portion of the gel stained with SYPRO Ruby to detect histones.

To verify which step of DNA replication was impaired in the MTBP-depleted extracts, we carried out chromatin isolation from MTBP-depleted, Treslin-depleted or mock-depleted extracts 90 min after the addition of sperm DNA to the extracts. In the MTBP- and Treslin-depleted extract, TopBP1 was recruited to chromatin, but the recruitment of RecQ4, Cdc45 and PCNA was strongly reduced (figure 4*e*,*f*). Since maximal loading of Treslin–MTBP requires both licensing and DDK activity and in addition to these TopBP1 loading requires CDK activity, we next determined the dynamic association of TopBP1 across the replication cycle in the absence of Treslin–MTBP. Figure 5*a* shows that TopBP1 chromatin association followed that of Treslin–MTBP, with a peak of binding during S phase which then declines as S phase proceeds. In extract depleted of Treslin–MTBP, the association of TopBP1 with chromatin was reduced but did not decline at later times. When either licensing or CDK activity were inhibited by the addition of geminin or p27^KIP1^, the association of TopBP1 during peak S phase in the absence of Treslin–MTBP was maintained though at a reduced level (figure 5*b,c*). In combination, these results suggest that although TopBP1 can associate with licensed chromatin in the presence of CDK activity, maximal binding during a normal S phase requires Treslin–MTBP. This extends previous reports of Treslin and MTBP depletion [23,38], and indicates that MTBP depletion impairs the assembly of the pre-IC required for the formation of the CMG helicase on chromatin.

**Figure 6.**
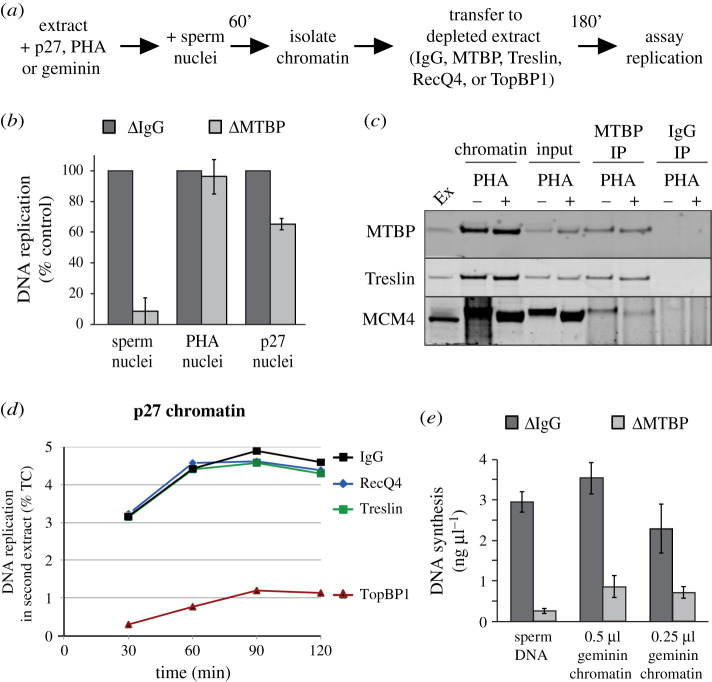
Treslin–MTBP complex regulation by replicative kinases. (*a*) Schematic of nuclear transfer experiments. (*b*) Sperm nuclei were incubated for 60 min in extract supplemented with p27^KIP1^ or PHA-767491; chromatin was isolated and transferred to extract immunodepleted with MTBP-1 antibodies or non-immune IgG; the extent of DNA synthesis after 180 min was determined. Average of three independent experiments; error bars represent standard error. (*c*) MTBP was immunoprecipitated from chromatin incubated in extract containing p27^KIP1^, in the presence or absence of PHA-767491 and samples were immunoblotted for the indicated proteins. (*d*) Sperm nuclei were incubated for 60 min in extract supplemented with p27^KIP1^; chromatin was isolated and transferred to extract immunodepleted with either Treslin-1, TopBP1, RecQ4 antibodies or non-immune IgG; the total DNA synthesis at the indicated times was determined. (*e*) Sperm nuclei were incubated for 60 min in extract supplemented with geminin; chromatin was isolated and transferred to extract immunodepleted with MTBP-1 antibodies or non-immune IgG; the total DNA synthesis after 180 min was determined. Average of three independent experiments; error bars represent standard error.

To determine whether the Treslin–MTBP complex loaded onto DNA in the absence of CDK or DDK activity is functional, we carried out chromatin transfer experiments (figure 6*a*). Sperm chromatin was incubated for 60 min in extract supplemented with p27^KIP1^ or PHA-767491 to inhibit CDK or DDK activities; the chromatin was then isolated and transferred to MTBP-depleted extract to determine if the previously loaded MTBP and Treslin could support replication initiation. As control, the same DNA templates were added to extract mock-depleted with non-immune IgG. Chromatin from extracts treated with PHA-767491 and p27^KIP1^ were able to replicate efficiently in MTBP-depleted extract, indicating that the Treslin–MTBP complex loaded onto chromatin in these conditions is potentially functional (figure 6*b*).

To verify whether the interaction between Treslin–MTBP and MCM2-7 is strengthened by DDK activity, as occurs in yeast [8], we treated interphase extract with p27^KIP1^ to block replication initiation and compared the amount of MCM co-precipitating with Treslin–MTBP plus or minus PHA-767491. Figure 6*c* shows that the amount of Mcm4 co-precipitating with Treslin–MTBP was lower when DDK activity was inhibited. Significantly, the residual Mcm4 co-precipitating in the presence of PHA-767491 was hyperphosphorylated compared to bulk chromatin, consistent with the idea that Treslin–MTBP binds more strongly to MCM2-7 that has been phosphorylated by DDK. Consistent with the ability of Treslin–MTBP and RecQ4 but not TopBP1 to associate with chromatin when CDK activity is inhibited, chromatin isolated from p27^KIP1^-treated extract could replicate efficiently in Treslin–MTBP and RecQ4 but not TopBP1-depleted extracts (figure 6*d*).

In order to determine whether the low quantity of Treslin–MTBP recruited to chromatin in the absence of licensing was functional, we isolated chromatin from extracts treated with geminin, which contained approximately 20% of control levels of Treslin–MTBP, and assayed its ability to replicate in MTBP-depleted extract. Figure 6*e* shows that the chromatin from geminin-treated extract replicated to less than 20% of control levels in MTBP-depleted extract. This suggests that origin licensing and MCM2–7 loading are required for Treslin–MTBP to be efficiently recruited to chromatin in a functional manner, but that the reduced amount of Treslin–MTBP loaded onto DNA in the absence of MCM2-7 is present in a potentially functional form.

### 2.6. DDK activity regulates Treslin–MTBP–TopBP1 complex formation

We have shown that both licensing and DDK activity are required both to increase and strengthen the association of Treslin–MTBP with chromatin. This is consistent with the hyperphosphorylation of Mcm4 that we have previously shown correlates with DNA replication [3,4]. The hyperphosphorylation of Mcm4 is reversed by Protein Phosphatase 1 (PP1) targeted to chromatin by Rif1. However, chromatin with hyperphosphorylated Mcm4 isolated from egg extract treated in the presence of I-2, an inhibitor of PP1, does not replicate fully when transferred to an extract in which both DDK and PP1 activity have been inhibited [3]. This suggests either that dephosphorylation of Mcm4 occurs even in the presence of I-2 or that there is a second DDK substrate required for the efficient initiation of DNA replication. In considering possible additional DDK substrates, we noted that chromatin-associated Treslin migrated slower in gels compared to the band present in extract, and that this slower migration was reduced when DDK activity was inhibited (figure 6*c*), suggesting that Treslin itself maybe a DDK substrate.

**Figure 7.**
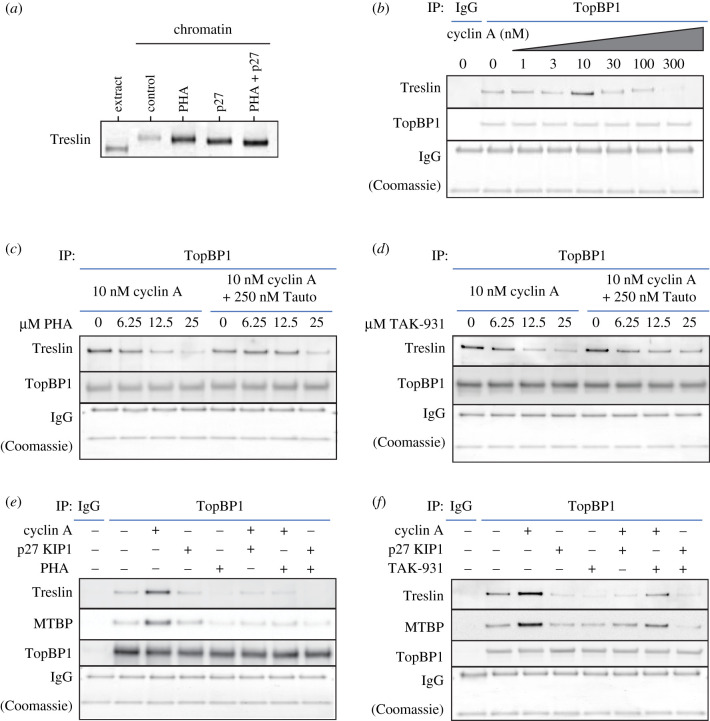
DDK and PP1 regulate CDK-mediated Treslin–MTBP and TopBP1 interaction. (*a*) Sperm nuclei were incubated in extract supplemented with either 100 nM p27^KIP1^ or 50 µM PHA-767491 alone or in combination, as indicated, for 60 min; chromatin was isolated, run on a NuPage gel and immunoblotted for Treslin. (*b–f*) Diluted and clarified egg extract was supplemented with cyclin A, p27^KIP1^, PHA-767491, TAK-931, Tautomycetin, either alone or in combination, as indicated and incubated for 15 min. Samples were immunoprecipitated using either control IgG or anti-TopBP1-antibody-bound protein-G Dynabeads. Washed precipitates were subjected to SDS–PAGE on a NuPage gel. The upper portion of the gel was immunoblotted for Treslin, MTBP and TopBP1 as indicated and the lower portion of the gel stained with Coomassie to visualize the heavy and light chains of IgG. For (*e*) and (*f*) 10 µM PHA-767491 or TAK-931, 100 nM p27 KIP1 and 10 nM cyclin A were used.

We, therefore, further characterized the change in Treslin migration in the presence of DDK inhibition by PHA-767491. We treated egg extract with inhibitors of either DDK or CDK activity alone or in combination, isolated chromatin following nuclear assembly during S phase and analysed Treslin mobility by immunoblotting. Figure 7*a* shows that upon inhibition of either DDK or CDK activity alone Treslin migrates faster than seen in control but to a different extent; in combination, inhibition of both DDK and CDK activity, results in Treslin migrating faster than in each of the treatments alone suggesting that both DDK and CDK independently contribute to the changes in Treslin mobility and that Treslin is a target of DDK activity.

Treslin–MTBP associates with chromatin-bound MCM2-7 and also forms a tripartite complex with TopBP1, thereby coupling replication licensing and pre-IC formation. We reasoned that since Treslin–MTBP associates more strongly with DDK-mediated hyperphosphorylated Mcm4 at licensed replication origins, DDK activity may also be required in addition to CDK activity to mediate Treslin–MTBP and TopBP1 interaction. We first determined the optimal level of CDK activity required to promote interaction between Treslin–MTBP and TopBP1. In the absence of nuclear formation, CDK activity in egg extract is low; upon nuclear formation and protein import, the local concentration of CDK activity within nuclei increases at least 25-fold, a level sufficient to induce replication initiation. In egg extract, the CDK1 catalytic subunit is in approximately 50-fold excess compared to its activating cyclin partners so addition of cyclin stimulates CDK activity *in vitro* [34]. To mimic the nuclear environment in which Treslin–MTBP–TopBP1 interaction occurs, we titrated cyclin A into fivefold diluted and clarified egg extract in the absence of DNA and assessed the formation of the Treslin–MTBP–TopBP1 complex by determining the recovery of Treslin in TopBP1 immunoprecipitates (figure 7*b*). The Treslin–TopBP1 interaction was maximal upon addition of 10 nM cyclin A into egg extract but declined thereafter as cyclin A concentration is further increased, concentrations at which the little Treslin that is seen to interact with TopBP1 is increasingly retarded in mobility. Maximal Treslin–TopBP1 interaction upon the addition of 10 nM cyclin A is consistent with the level of Cdk2–cyclin A required to support maximal DNA replication in whole egg extracts, above which the extract enters a mitotic-like state [34].

We next determined the effect of the addition of inhibitors of DDK to egg extract on cyclin A-stimulated Treslin–TopBP1 interaction. Figure 7*c*,*d* shows that the addition of the DDK inhibitors PHA-767491 or TAK-931 reduced the interaction of Treslin with TopBP1. TAK-931 (simurosertib) is a recently identified thieno-pyrimidinone-based inhibitor of Cdc7 [43]. Electronic supplementary material, figure S8a–c shows that compared to PHA-767491, TAK-931 is a more specific but marginally less potent inhibitor of DNA replication and DDK-mediated chromatin-associated Mcm4 phosphorylation in egg extract. Like PHA-767491 (figure 7*a*), addition of TAK-931 to extract enhances the mobility of Treslin on gels (electronic supplementary material, figure S8d), consistent with Treslin being phosphorylated in a DDK-dependent manner. Furthermore, figure 7*c*,*d* shows that both PHA-767491 and TAK-931-mediated inhibition of Treslin–TopBP1 interaction was partially reversed upon co-addition of the PP1 inhibitor Tautomycetin into extract, consistent with the role of PP1 in reversing DDK-mediated phosphorylation of Mcm4 [3,4]. Similar (though less strong) inhibition was obtained with a third DDK inhibitor, XL413 (electronic supplementary material, figure S9a). These results suggest that DDK activity is required for the interaction of Treslin–MTBP and TopBP1 and that this is subject to regulation by PP1.

Whereas CDK activity in the absence of nuclear formation is low in egg extract, DDK activity is detectable but reduced [3,4]. To further address the role of DDK activity on the formation of the Treslin–MTBP–TopBP1 complex, we next determined the effect of combined inhibition of both DDK and CDK activity on their interaction. Figure 7*e*,*f* shows that cyclin A's ability to stimulate formation of the Treslin–MTBP–TopBP1 complex is curbed both by inhibiting CDK activity with p27^KIP1^ and by inhibiting DDK activity with either PHA-767491 or TAK-931. The inhibition of both CDK and DDK activities reduced complex formation to the greatest extent but no more so than with DDK inhibitor alone. Similar though less strong inhibition was obtained with XL413 (electronic supplementary material, figure S9b). This suggests both that the formation of the Treslin–MTBP–TopBP1 complex is dynamic and that DDK activity is required for the CDK-dependent formation of the complex.

**Figure 8.**
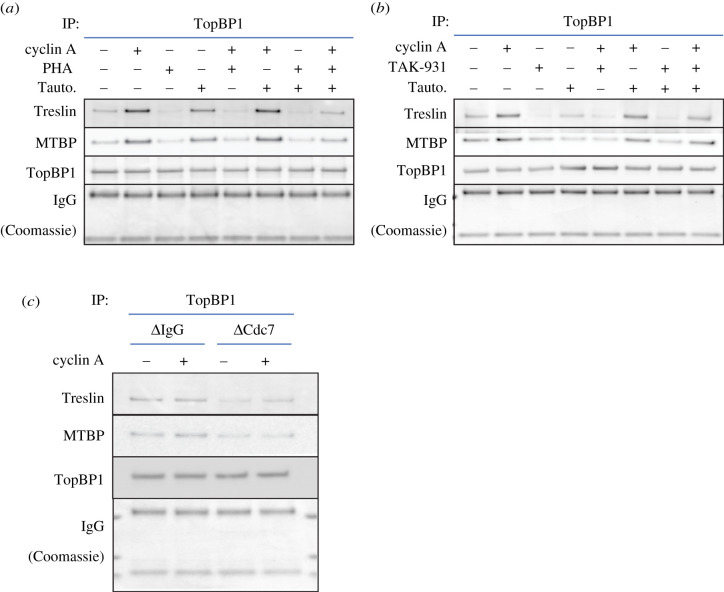
PP1 reverses DDK-mediated inhibition of CDK-mediated Treslin–MTBP and TopBP1 complex formation. (*a*,*b*) Diluted and clarified egg extract was supplemented with either 10 nM cyclin A, 100 nM p27^KIP1^, 10 µM PHA-767491, 10 µM TAK-931, 250 nM Tautomycetin, either alone or in combination, as indicated and incubated for 15 min. Samples were immunoprecipitated using either control IgG or anti-TopBP1-antibody-bound protein-G Dynabeads. Washed precipitates were subjected to SDS–PAGE on a NuPage gel. The upper portion of the gel was immunoblotted for Treslin, MTBP and TopBP1 as indicated and the lower portion of the gel stained with Coomassie to visualize the heavy and light chains of IgG. (*c*) Extract immunodepleted with a control IgG or anti-Cdc7 (DDK) antibody was either supplemented or not with 10 nM cyclin A, as indicated and incubated for 15 min. Samples were immunoprecipitated using either control IgG or anti-TopBP1-antibody-bound protein-G Dynabeads. Washed precipitates were subjected to SDS–PAGE on a NuPage gel. The upper portion of the gel was immunoblotted for Treslin, MTBP and TopBP1 as indicated and the lower portion of the gel stained with Coomassie to visualize the heavy and light chains of IgG.

The dynamic nature of the Treslin–MTBP–TopBP1 interaction is consistent with the role we identified for PP1 in reversing DDK inhibition of complex formation (figure 7*c,d*). Maximal interaction required both optimal CDK and DDK activity. Figure 8*a,b* shows that addition of the PP1 inhibitor Tautomycetin alone stimulated the interaction between Treslin–MTBP and TopBP1, although not to the same extent as the addition of cyclin A. Maximal complex formation was seen when both DDK and CDK activity were stimulated by the combined addition of both Tautomycetin and cyclin A. Whereas addition of either PHA-767491 or TAK-931 reversed the stimulation seen upon addition of Tautomycetin, the further addition of cyclin A could overcome this to some extent. These results are consistent with the idea that DDK activity is actually more important than CDK activity for the formation of the Treslin–MTBP–TopBP1 complex. Complex formation was more strongly reduced by DDK inhibition than by CDK inhibition (figure 7*e,f*). Stimulation of DDK-dependent phosphorylation with tautomycetin promoted complex formation in the absence of cyclin A, but only very low levels of complex formation occurred when DDK activity was inhibited in the presence of cyclin A. Similar though less strong inhibition was obtained with XL413 (electronic supplementary material, figure S9c). Taken together, these results suggest that DDK activity is essential for the formation of the Treslin–MTBP–TopBP1 complex, while optimal CDK activity further strengthens the interactions.

To address directly the role of DDK activity in Treslin–MTBP–TopBP1 complex formation, we next prepared extract immunodepleted of Cdc7. Immunodepletion of at least 95% of Cdc7 from the extract left MTBP levels similar to those in control depleted extract (electronic supplementary material, figure S10a). Consistent with the results presented above using both PHA-767491 and TAK-931 to inhibit DDK activity, removal of Cdc7 from egg extract reduced the interaction of Treslin–MTBP with TopBP1 both in the presence and absence of additional cyclin A (figure 8*c*; electronic supplementary material, figure S10b).

## 3. Discussion

In this study, we show that in *Xenopus* egg extracts, MTBP and Treslin form an elongated complex that is recruited to DNA before replication initiates. The Treslin–MTBP complex can be recruited to DNA in a functional form in the absence of replication licensing, DDK or CDK activity, but licensing and DDK activity stabilizes the interaction between Treslin–MTBP and chromatin-bound MCM2-7. We find that the Treslin–MTBP complex is present in extract at a low concentration expected to be rate limiting for replication initiation. We also demonstrate an important and previously unrecognized role for DDK activity in promoting the interaction of Treslin–MTBP with TopBP1. Since DDK also has an essential role in phosphorylating chromatin-bound MCM2-7, this suggests that DDK plays a central role in directing the recruitment of Treslin–MTBP and TopBP1 to licensed replication origins, thereby selecting which origins will subsequently initiate.

Treslin and MTBP co-immunoprecipitate with each other in both metaphase and interphase extract indicating that they interact throughout the cell cycle, as previously reported in human cells and *Xenopus*, and similar to Sld3 and Sld7 in budding yeast [13,19,23]. Using a combination of sucrose gradient sedimentation and gel filtration, we show that the Treslin–MTBP complex has a native molecular weight of 626 kDa, close to (± less than or equal to1%) the value expected for a tetramer composed of two molecules of MTBP (96 kDa) and two molecules of Treslin (220 kDa), that is 632 kDa. The distinct behaviours of *Xenopus* Treslin–MTBP on gel filtration and sucrose gradients suggest that it forms a highly elongated complex. This result is in line with the model proposed for Sld3 and Sld7 in yeast—obtained from studies of the crystal structures of domains of Sld3 and Sld7—which proposed that two molecules of Sld7 could bridge two molecules of Sld3, with one loaded onto each of the hexamers of MCM2-7 loaded at an origin to allow bidirectional initiation [14].

MTBP and Treslin also interact on chromatin during S phase and at this time they also interact with Cdc45 and the GINS complex, consistent with the idea that they function during replication initiation. Immunodepletion of MTBP from *Xenopus* egg extract co-depleted Treslin with impairment of DNA replication due to the lack of CMG assembly, supporting the idea that Treslin–MTBP is required for CMG formation. The capacity to initiate replication could be restored to MTBP-depleted extract by co-addition of a partially purified Treslin–MTBP complex, but not by fractions containing MTBP alone. This is consistent with a recent report which shows that DNA replication in an MTBP-depleted extract can be rescued with a combination of recombinant Treslin and MTBP, but not either alone [23].

**Figure 9.**
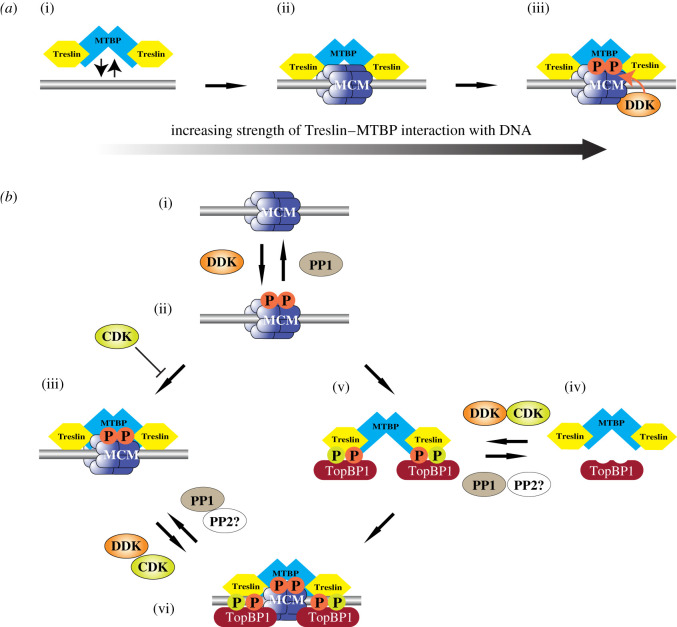
Model for the role of Treslin–MTBP, DDK and CDK in pre-IC formation. (*a*) Model of the interaction of Treslin–MTBP with replication origins. (i) Treslin–MTBP can bind weakly to DNA. (ii) Stronger binding of Treslin–MTBP to licensed origins (DNA-bound MCM2–7 double hexamers). (iii) Strongest binding of Treslin–MTBP to licensed origins where MCM2–7 has been phosphorylated by DDK. (*b*) Model for pre-IC formation. (i) Licensed origin (DNA-bound MCM2–7 double hexamers). (ii) DNA-bound MCM2–7 is phosphorylated by DDK. Phosphorylation is reversed by PP1 recruited to origins by Rif1. (iii) Treslin–MTBP is recruited to DDK-phosphorylated MCM2–7. This interaction is opposed by CDK, probably due to CDK phosphorylation of Treslin. (iv) Soluble Treslin bound to MTBP can be phosphorylated by both CDK and DDK. (v) Phosphorylated Treslin–MTBP binds to TopBP1. (vi) The Treslin–MTBP–TopBP1 complex is recruited to DDK-phosphorylated MCM2–7 to form the pre-IC.

We found that MTBP was recruited onto chromatin during licensing and peaked in S phase, similar to Treslin. The chromatin recruitment of MTBP and Treslin occurred in the absence of licensing, DDK, CDK or nuclear assembly (figure 9*a*(i)), but did not occur in metaphase-arrested extract. Chromatin transfer experiments showed that the Treslin–MTBP recruited onto chromatin in the absence of licensing, DDK or CDK could efficiently support replication in MTBP-depleted extract; this suggests that Treslin–MTBP binds unlicensed chromatin in a form that can be efficiently activated when other initiation activities are supplied. These results differ from studies in yeast, where the loading of Sld3 and Sld7 onto origins depends on licensing and DDK activity [7]. However, we show that in extract where licensing was abolished by addition of active geminin or RL5a, the loading of MTBP and Treslin onto chromatin was strongly reduced compared to control, indicating that the MTBP and Treslin loading that occurs before licensing is relatively unstable (figure 9*a*(ii)). MCM2–7 is known to be phosphorylated by DDK and this phosphorylation is required for pre-IC formation [1–4,44]. We show here that consistent with data in yeast [8] the physical interaction between Treslin–MTBP and MCM2–7 was strengthened by DDK activity (figure 9*a*(iii)). The Treslin–MTBP bound to chromatin in the absence of either licensing or DDK and CDK activity can support DNA replication, suggesting that it is in a potentially active configuration.

Whereas both licensing and DDK activity are required to both increase and strengthen the interaction of Treslin–MTBP with chromatin, CDK activity reduces this interaction (figure 9*b*(i–iii)). Treslin is a CDK substrate and interacts with TopBP1 through its BRCT motifs following CDK-mediated phosphorylation of Treslin S1001 [20,38,45]. It is therefore likely that CDK-dependent phosphorylation of Treslin reduces its affinity for MCM2–7 while increasing its affinity for TopBP1. Therefore, as CDK activity rises during S phase, Treslin–MTBP is likely to be recruited to replication origins as part of a complex with TopBP1 (figure 9*b*(iv,v)) and that this is a precursor to the formation of the pre-IC (figure 9*b*(vi)). We find that, in addition to the known regulation of the Treslin–TopBP1 interaction by CDK, DDK activity also plays a major role (figure 9*b*(v,vi)). The inhibition of DDK activity either by the addition of PHA-767491, TAK-931 or XL413 or by depletion of Cdc7 restricts the interaction between Treslin–MTBP and TopBP1, even in the presence of an optimal concentration of cyclin A to support complex formation, identifying the DDK dependence of CDK-mediated complex formation. In addition, we find that the DDK-mediated regulation of complex formation is subject to reversal by PP1. By increasing and decreasing DDK and CDK activity separately, we provide data suggesting that DDK activity is essential for the formation of the Treslin–MTBP–TopBP1 complex, while optimal CDK activity further strengthens the interactions.

Given that Treslin–MTBP binds to chromatin prior to nuclear assembly when DDK activity is detectable but reduced and before CDK activity rises to support replication initiation, it may be that TopBP1 is initially directed to Treslin–MTBP associated with licensed and DDK-phosphorylated origins, forming a complex directly at the origin (figure 9*b*(i–iii)), but that as CDK activity rises during S phase, the Treslin–MTBP–TopBP1 complex plays a major role in driving formation of the pre-IC (figure 9*b*(vi–vi)). This suggests that in addition to ensuring Treslin–MTBP–TopBP1 complex formation, CDK activity facilitates the removal of Treslin–MTBP from unlicensed chromatin, thus ensuring redistribution of the complex to only licensed and DDK-phosphorylated origins to maximize the levels of origin firing. DDK activity thus coordinates the coupling of replication licensing to pre-IC formation and therefore, ultimately, CMG formation, by both directing the correct chromatin binding of Treslin–MTBP and mediating its interaction with TopBP1.

Studies in *S. cerevisiae* suggest that Sld3, Sld2, Dpb11 and Dbf4 are present at limiting concentrations [12,13,46]. Moreover, overexpression of these factors together with overexpression of Sld7 and Cdc45 increased the efficiency of origin firing, including the firing of normally dormant origins [46]. In *Xenopus* embryos, it has been found that Treslin, TopBP1, Drf1 and RecQ4 are limiting for replication after the mid-blastula transition and the levels of these factors regulate the speed of cell division [47]. We quantified the amount of MTBP in *Xenopus* egg extract and found that the concentration of the Treslin–MTBP tetramer is approximately 1 nM. This is significantly lower than most other replication proteins and is lower than the estimated 4 nM of replication origins that can be efficiently replicated in *Xenopus* embryos and egg extracts [26,27]. Consistent with this, we showed that the replication rate in extract is highly sensitive to the concentration of Treslin–MTBP. This is also consistent with a previous report showing that the majority of Treslin becomes chromatin-bound as embryos approach the mid-blastula transition [47]. The low concentration of Treslin–MTBP in embryos is therefore likely to be rate limiting for replication initiation as embryos approach the mid-blastula transition and DNA concentration approaches the maximum capacity observed in egg extracts.

In yeast, Sld3 is required for replication initiation but is not part of the replisome and is not required for replication fork progression [48,49]. The chromatin binding of *Xenopus* Treslin–MTBP supports this idea, as Treslin–MTBP is lost from chromatin in the later part of S phase when some replisomes are still active. As an initiation activity, Treslin–MTBP, possibly also associated with TopBP1, is therefore expected to be released from origins as they initiate, potentially being able to bind again to unfired origins to promote their initiation. At the maximum physiological concentration of DNA in *Xenopus* egg extract (close to the DNA concentration at the mid-blastula transition), the concentration of active origins is approximately 4 nM [26,27]. We estimate approximately 1 nM of the Treslin–MTBP tetramer in egg extract, meaning that each molecule would have to drive the initiation of an average of four origins at this maximum DNA concentration. This is consistent with previous work on the kinetics of origin firing in *Xenopus* extract which estimated that S phase is driven by approximately 3 cycles of origin firing [50]. Furthermore, given the known role of the inhibition of DDK activity in checkpoint signalling following the interruption of the progression of DNA replication, the identification of DDK and PP1 as regulators of Treslin–TopBP1 complex formation provides a novel point at which DNA replication could be regulated following checkpoint activation.

Not all licensed replication origins initiate in any given S phase, with the majority remaining dormant but able to initiate in the case of replication stresses [51–55]. In *Xenopus* egg extracts, less than 10% of DNA-bound MCM2–7 initiate in an undisturbed S phase [51,56,57], meaning that at mid-blastula transition DNA concentrations, the concentration of DNA-bound MCM2–7 is at least 50 nM. Given the data we present here, it is highly likely that Treslin–MTBP plays a crucial role in determining which of the licensed origins are selected for initiation. In egg extract, there is high DDK activity so that almost all MCM2–7 loaded onto DNA become phosphorylated by DDKs [3,4]. The loading of the limited amounts of Treslin–MTBP is the earliest known event in the initiation process that selects the origins that will go on to initiate, as the other limiting factors (TopBP1 and RecQ4) act after Treslin–MTBP has been loaded. The correct selection of replication origins is important for maintaining genome stability by regulating the distribution of active replication forks in response to replication stresses [52,54,55]. We therefore predict that over- or under-expression of Treslin–MTBP causes defects in correct origin selection, providing an explanation for why MTBP is misregulated in certain cancers [18].

Our data show that DDK directs the initiation of DNA replication at individual origins by two distinct means: (i) priming potential replication origins for activation by phosphorylating MCM2–7 to promote Treslin–MTBP association and therefore pre-IC assembly, and (ii) directing formation of the Treslin–MTBP–TopBP1 complex that selects these primed origins for activation. With PP1 able to reverse both of these DDK-mediated activities, the local balance of DDK : PP1 activity, together with that of CDK, must therefore be coordinated to control origin activation. The potential role of a phosphatase such as PP2A to reverse the CDK-mediated phosphorylation of Treslin required for Treslin–MTBP–TopBP1 complex formation remains to be determined. That Treslin–MTBP–TopBP1 complex formation requires the integration of the two S phase kinases, CDK and DDK, potentially allows CDK to act as the ‘global’ regulator of S phase progression, while ‘local’ control of the initiation of DNA replication is delegated to DDK activity.

## 4. Methods

### 4.1. Contact for reagent and resource sharing

Requests for resources, reagents and further information should be directed to and will be fulfilled by the corresponding author.

### 4.2. Experimental model and subject details

#### 4.2.1. Xenopus laevis

Wild-type, sexually mature (at least 1 year old) female and male South African clawed frogs (*Xenopus laevis*) born and reared in the UK (University of Plymouth) were used in this study for the production of unfertilized eggs (from which extracts were prepared) and sperm, respectively. Frogs were maintained at 19°C in particulate filtered, dechlorinated water, at a density of less than or equal to 15 animals per 60 l tank, in a purpose built ‘aquacentre’ and were maintained by professional staff at the University of Dundee adhering to Home Office (UK Government) animal husbandry guidelines; the animals have access to a Home Office (UK Government) approved veterinary surgeon. The frogs were fed a vegetable and fish-based diet (Aquatic Diets 3, Mazuri Zoo Foods) 2–3 times per week, as required.

### 4.3. Experimental procedures

#### 4.3.1. Recombinant antigens, antibodies and inhibitors

Polyclonal antibodies were raised in rabbit against the following polypeptides: MTBP-2: 125 N-terminal amino acids of MTBP with 6-histidine tag at C-terminus (MERFVLCIHWERRAEQQQPVPQGLVYAQDIYTQLKEYSTNCTSTFPACSLTGNPGIRKWFFALQSLYGFSQFCSSDWEDLCPAVTTDDSEEPVQTALDECLDALQFPDGEDDNSRDSISQTNLFE) MTBP-1: 103 C-terminal amino acids of MTBP with 6-histidine tag at N-terminus (APSVPAQSKPSSHLELEQKESRSQKHNRMLKEVVSKTLQKHSIGVEHPCYAACNQRLFEISKFFLKDLKTSRGLLDEMKKAASNNAKQVIQWELDKLKKK). Antibodies were purified using rProtein-A Sepharose fast flow beads (Cytiva LifeSciences) or affinity purified using HiTrap NHS-Activated HP columns (Cytiva LifeSciences). For Treslin, polyclonal antibodies were raised in two different rabbits against the N-terminal 487 amino acids of *Xenopus* Treslin, with an N-terminal 6-histidine tag. For TopBP1, polyclonal antibodies were raised in sheep against the N-terminal 596 amino acids of *Xenopus* TopBP1, with an N-terminal 6-histidine tag.

Commercial antibodies used in this study are: anti-histone H3 FL-136 rabbit (Santa Cruz; sc-10809); anti-hMTBP (MTBP-S: Sigma-Aldrich HPA025694); anti-hPCNA (PC10: Santa Cruz; sc-56). Antibodies against *Xenopus* proteins: Cdc45 and Sld5 [58]; Mcm4 [59]; Orc1 [60]; Orc2 [61]. Anti-*Xenopus* WRN rabbit polyclonal antibody was a gift from H. Yan (Fox Chase Cancer Center, Philadelphia, USA) [62]. Anti-rabbit IgG, anti-sheep IgG and anti-mouse–HRP-linked antibodies were from New England Biolabs. Anti-rabbit IgG, anti-sheep IgG and anti-mouse IgG–Dylight 800 or Dylight 680 linked antibodies were from ThermoFisher Scientific.

Recombinant geminin^DEL^ was as described [63]. Recombinant p27^KIP1^ was as described [3]. Recombinant C′ terminal HIS-tagged bovine ΔN-cyclin A (187–419) was purchased from MRC PPU Reagents & Services (DU12276). PHA-767491 was produced by Division of Signal Transduction Therapy, University of Dundee, TAK-931 was purchased from Insight Biotech (HY-10088) and XL413 was purchased from Axon Medchem (no. 2268). *Triticum vulgaris* WGA was purchased from Sigma-Aldrich (L9640).

#### 4.3.2. *Xenopus* egg extract methods

Metaphase-arrested *X. laevis* egg extract and demembranated *Xenopus* sperm nuclei were prepared as described [24,64]. Female frogs were primed with 50 units of Folligon (Pregnant Mare Serum Gonadotrophin) 3 days before the eggs were required to increase the number of stage 6 mature oocytes and 2 days later, were injected with 500 units Chorulon (Chorionic Gonadotrophin) to induce ovulation. Frogs were placed in individual laying tanks at 18–21°C in 2 l 1× MMR egg laying buffer, prepared from a 10× stock (1 M NaCl, 20 mM KCl, 10 mM MgCl_2_, 20 mM CaCl_2_,1 mM EDTA, 50 mM HEPES NaOH, pH 7.8). The following morning, eggs were collected and rinsed in 1× MMR to remove any non-egg debris. Washed eggs were dejellied in 2% w/v cysteine (pH 7.8), washed in XBE2 (1× XB salts, 1.71% w:v sucrose, 5 mM K-EGTA, 10 mM HEPES KOH, pH 7.7; 10× XB salts: 2 M KCl, 40 mM MgCl_2_, 2 mM CaCl_2_) and then into XBE2 containing 10 µg ml^−1^ leupeptin, pepstatin and aprotinin. Dejellied and washed eggs were centrifuged in 14 ml tubes, containing 1 ml XBE2 plus protease inhibitors containing 100 µg ml^−1^ cytochalasin D, at 1400*g* in a swinging bucket rotor for 1 min at 16°C to pack the eggs, after which excess buffer and dead eggs were removed. Packed eggs were crushed by centrifugation at 16 000*g* in a swinging bucket rotor for 10 min at 16°C. The dirty brown cytoplasmic layer was collected using a 20 G needle and a 1 ml syringe via side puncture. From this point onwards, the extract was kept on ice. The crude extract was supplemented with cytochalasin D, leupeptin, pepstatin and aprotinin all to a final concentration of 10 µg ml^−1^, 1 : 80 dilution of energy regenerator (1 M phosphocreatine disodium salt, 600 µg ml^−1^ creatine phosphokinase in 10 mM HEPES KOH, pH 7.6) and 15% v:v LFB1/50 (10% w:v sucrose, 50 mM KCl, 2 mM MgCl_2_, 1 mM EGTA, 2 mM DTT, 20 mM K_2_HPO_4_/KH_2_PO_4_ pH 8.0, 40 mM HEPES KOH, pH 8.0). The extract was clarified by centrifugation at 84 000*g* in a pre-cooled SW55 swinging bucket rotor at 4°C for 20 min. The golden cytoplasmic layer was recovered, supplemented with glycerol to 2% v/v and frozen in aliquots in liquid nitrogen and stored at −80°C until required.

Sperm was recovered from testes isolated from male frogs post-mortem following a lethal dose of anaesthetic (0.2% w:v Tricaine mesylate MS222, approx. 0.5% w:v NaHCO_3_ to pH 7.5). Isolated testes were washed carefully to avoid bursting in EB (50 mM KCl, 5 mM MgCl_2_, 2 mM dithiothreitol or β-mercaptoethanol, 50 mM HEPES KOH, pH 7.6), prior to being finely chopped with a clean razor blade in fresh EB. Recovered lysate was filtered through a 25 µm nylon membrane to remove particulate matter. Filtered sperm was centrifuged at 2000*g* at 4°C for 5 min; selective resuspension of the sperm pellet allowed separation of the sperm from contaminating erythrocytes; the resuspended sperm was respun and the pellet resuspended in 0.5 ml SuNaSp (0.25 M sucrose, 75 mM NaCl, 0.5 mM spermidine, 0.15 mM spermine, 15 mM HEPES KOH, pH 7.6) per testis. The sperm was demembranated with the addition of 25 µl per testis lysolecithin (5 mg ml^−1^, in H_2_O) for 10 min at room temperature. Demembranated sperm were respun and resuspended in SuNaSp plus 3% w/v BSA to quench the demembranation reaction. Quenched sperm were respun and resuspended in 100 µl EB plus 30% glycerol per testis, counted using a haemocytometer and stored at −80°C.

Extracts were supplemented with 250 µg ml^−1^ cycloheximide, 25 mM phosphocreatine and 15 µg ml^−1^ creatine phosphokinase and incubated with 0.3 mM CaCl_2_ for 15 min to trigger release from metaphase arrest. For DNA synthesis reactions, demembranated *Xenopus* sperm nuclei were incubated at 3–10 ng DNA µl^−1^ in extract. DNA synthesis was assayed by measuring incorporation of [α-^32^P] dATP into acid-insoluble material followed by scintillation counting, as described [24,64]. Extract was supplemented with 50 nCi µl^−1^ [α-^32^P] dATP from a high activity 10 mCi ml^−1^ stock. At the appropriate times, 10 µl aliquots was stopped by the addition of 160 µl Stop-C (0.5% w:v SDS, 5 mM EGTA, 20 mM Tris HCl, pH 7.5) plus freshly added 0.2 mg ml Proteinase K (from stock of 20 mg ml^−1^ proteinase K, 50% v:v glycerol, 10 mM Tris–HCl, pH 7.5) and were incubated at 37°C for 30 min. Samples are precipitated at 4°C for 30 min by the addition of 4 ml 10% TCA (10% w:v TCA, 2% w:v Na_4_P_2_O_7_·10H_2_O). Forty microlitres (1% of 4 ml) of the total reaction were spotted on a paper disc. Insoluble material was recovered from solution by filtration through a glass fibre filter mounted on a vacuum manifold. The glass fibre filters were twice washed in 5% TCA (5% w:v TCA, 0.5% w:v Na_4_P_2_O7.10H_2_O), once in 100% ethanol and then air dried. The paper and glass fibre filters were then quantified by scintillation counting. Precipitated material was expressed as a percentage of total counts (%TC) from which DNA replication (ng µl^−1^) was calculated by multiplying by a factor of 0.654. The extent of nuclear formation was followed under the light microscope (phase contrast). All incubations were carried out at 23°C.

Licensing factor extract (LFE) was prepared as described [65]. The initial steps for preparing metaphase extracts were followed. Before the final spin, the extract was activated with 0.3 mM CaCl_2_ for 15 min then diluted fivefold with ‘Licensing Factor Buffer’ (LFB1: 40 mM HEPES KOH pH 8.0, 20 mM K_2_HPO_4_/KH_2_PO_4_ pH 8.0, 2 mM MgCl_2_, 1 mM EGTA, 2 mM DTT, 10% (w/v) sucrose and 1 µg ml^−1^ each of leupeptin, pepstatin and aprotinin) supplemented with 50 mM KCl (i.e. LFB1/50) and spun to remove membrane components at 84 000*g* in a pre-cooled SW55 rotor swinging bucket rotor at 4°C for 40 min. The clarified supernatant was frozen, in aliquots, in liquid nitrogen and stored at −80°C till required.

Membrane-free extract (MFE) used for TopBP1 immunoprecipitation experiments was prepared as per LFE, above, but was spun at 186 000*g* in a pre-cooled TLA-100 fixed angle rotor at 4°C for 20 min and recovered supernatant was used immediately.

Immunodepleted extract was prepared as described [65]. Briefly, rProtein-A agarose beads (Cytiva LifeSciences), preincubated with 2 volumes of serum, were twice incubated with interphase whole egg extract at a ratio of 1 volume extract plus 0.7 volume beads. Twice depleted extract, recovered from the beads, was frozen in aliquots in liquid nitrogen and stored at −80°C.

Immunoprecipitations using affinity purified antibody saturated Protein A and Protein-G Dynabeads were performed following the manufacturer's instructions (ThermoFisher Scientific).

#### 4.3.3. Chromatin isolation

All chromatin isolations from *Xenopus* egg extracts were undertaken in low-adhesive Eppendorf tubes, as described [24,64]. Briefly, reactions were stopped by the addition of 400 µl of ice-cold NIBTX (50 mM KCl, 50 mM HEPES KOH pH 7.6, 5 mM MgCl_2_, 2 mM DDT, 0.5 mM spermidine 3HCl, 0.15 mM spermine 4HCl, 0.1% Triton X-100). This was underlayered with 100 µl 15% sucrose in NIBTX. The tubes were spun at 6000*g* for 5 min at 4°C in a swinging bucket rotor. The buffer above the sucrose cushion was removed and the surface of the cushion washed with 200 µl NIBTX before removing the cushion down to approximately 15 µl. The tubes were then spun at 13 000*g* for 2 min in a fixed angle rotor to focus the chromatin pellet and following this, all the buffer was removed. The chromatin pellet was then resuspended in loading buffer and was subjected to immunoblotting by standard techniques using 4–12% Bis–Tris gradient SDS–PAGE gel (Invitrogen) with the exception of figure 7*a* for which a 3–8% Tris–Acetate gel (Invitrogen) was used.

For nuclear transfer experiments, 90 µl interphase extract supplemented with appropriate inhibitors was incubated with 26 ng µl^−1^ of sperm nuclei for 1 h. Extract was diluted with 1 ml of NIB50 buffer (50 mM KCl, 50 mM HEPES KOH pH 7.6, 5 mM MgCl_2_, 2 mM DDT, 0.5 mM spermidine 3HCl, 0.15 mM spermine 4HCl) without Triton X-100, underlaid with 100 µl NIB50 containing 15% sucrose, and 5 µl NIB50 containing 30% glycerol, and spun at 2100*g*, 5 min at 4°C in a swinging bucket rotor bench centrifuge. The sucrose cushion was washed with 200 µl NIB50, the buffer was removed leaving approximately 15 µl. Nuclei were gently resuspended and frozen in liquid nitrogen.

For the geminin chromatin transfer assay [66], 80 µl of extract were activated and incubated for 5 min with 120 nM recombinant geminin^DEL^, 20 ng µl^−1^ of demembranated sperm nuclei were added to the extract and incubated for 12 min. Chromatin was isolated by diluting the reaction with 1 ml of NIB50, underlaid with 100 µl of NIB50 containing 15% sucrose, underlaid with 5 µl of NIB50 containing 30% glycerol and spun at 6000*g*, 5 min at 4°C in a swinging bucket rotor bench centrifuge, all in the absence of Triton X-100. The buffer above the sucrose cushion was washed with 200 µl NIB50 without Triton and the buffer was removed leaving approximately 15 µl sample. Chromatin was gently resuspended by inversion and frozen in aliquots in liquid nitrogen.

#### 4.3.4. Immunoblotting

Samples were prepared in 6× Laemmli sample buffer heated at 99°C for 4 min and separated on 4–12% Bis–Tris NuPage gels (Invitrogen) in 1× MOPS running buffer (Invitrogen) or 3–8% Tris–Acetate gels (Invitrogen) in Tris–Acetate running buffer (Invitrogen). Proteins were transferred onto nitrocellulose membrane (Cytiva LifeSciences) and images captured using an Odyssey SLX LiCor Scanner (Licor Bioscience) when using fluorescently labelled secondary antibodies or transferred onto PVDF (Cytiva LifeSciences) when detected using enhanced chemiluminescence detection (Pierce SuperSignal West Pico Chemiluminescent Substrate 34087; ThermoFisher Scientific) and images captured using an ImageQuant LAS4000 (Cytiva LifeSciences) CCD. Quantification of MTBP was carried out using pre-diluted Bovine Gamma Globulin protein assay standards (Pierce 23213; ThermoFisher Scientific) and analysed using Image Studio Lite LICOR software.

For protein visualization, gels were stained with either SYPRO Ruby (ThermoFisher Scientific) and de-stained with 10% ethanol and 7% acetic acid in H_2_O or with Coomassie R-250 and de-stained with 40% ethanol and 10% acetic acid in H_2_O.

#### 4.3.5. Mass spectrometry

Immunoprecipitated samples for mass spectrometry were run on NuPage gels and stained with SimplyBlue SafeStain (Invitrogen). Bands at the size of Treslin and MTBP were cut from gels and reduced with dithiothreitol, alkylated with iodoacetamide and in-gel digested with trypsin. The extracted peptide solutions were analysed using nano LC-MS/MS on an LTQ Orbitrap Velos (ThermoFisher Scientific).

#### 4.3.6. Gel filtration and glycerol gradients

For gel filtration, 100 µl of a 3% PEG precipitate resuspended in LFB1/50 to a final volume of twice that of undiluted extract was applied to an Agilent BIO SEC-5 column (500 Å 4.6 × 300 mm, range 15–5000 kDa) equilibrated in LFB1/50. The column was run at a flow rate of 150 µl min^−1^ on an UltiMate 3000 chromatography system (ThermoFisher Scientific) at 4°C and 75 µl fractions were collected. For sucrose gradient centrifugation, 440 µl of a 3% PEG precipitate resuspended in LFB1/50 to a final volume of twice that of undiluted extract was applied to an 11 ml 5–40% sucrose gradient prepared in LFB1/50. Gradients were spun at 40 000 rpm (approx. 111 000*g*) for 20 h in a SW41Ti swinging bucket rotor in a Beckman Optima ultracentrifuge. Four hundred and forty microlitre fractions were collected.

Molecular masses were calculated according to Siegel & Monty [25] using the values: thyroglobulin tetramer (1338 kDa, 107 Å), thyroglobulin dimer (669 kDa, 85 Å, 19.5 S), apoferritin (443 kDa, 67 Å, 17.6 S), β-amylase (200 kDa, 54 Å, 8.9 S), BSA (66 kDa, 35.5 Å, 4.3 S), carbonic anhydrase (29 kDa, 24.3 Å, 3.2 S) (Sigma-Aldrich MWGF1000).

#### 4.3.7. Partial purification of Treslin–MTBP

PEG precipitation was carried out as described [65]. Briefly, to one volume of LFE, the required volume of 50% v:v of PEG 6000 in H_2_O was added, mixed well and incubated for 30 min on ice-water. The samples were spun at 20 000*g*, 30 min at 4°C in a fixed angle rotor. The supernatant was removed and the pellet resuspended in the required amount of LFB1/50 (50 mM KCl, 20 mM K_2_HPO_4_/KH_2_PO_4_ pH 8.0, 40 mM HEPES KOH pH 8.0, 2 mM MgCl_2_, 1 mM EGTA, 2 mM dithiothreitol, 10% (w:v) sucrose).

SP and ANX HiTrap 1 ml columns (Cytiva LifeSciences) were washed with 5 column volumes of H_2_O, 5 column volumes of LFB1/50, 5 column volumes of LFB1 containing 1 M KCl and 10 column volumes of LFB1/50. Samples were spun at 21 910*g* for 20 min at 4°C in a fixed angle rotor in a benchtop centrifuge prior to column loading. Columns were connected to an AKTApurifier UPC10 system (Cytiva LifeSciences) or to an UltiMate3000 chromatography system (ThermoFisher Scientific). When the OD_280_ was flat, the proteins bound to the matrix of the columns were eluted through a gradient from 50 to 750 mM KCl in 14 × 1 ml fractions at a flow rate of 1 ml min^−1^.

#### 4.3.8. Quantification and statistical analysis

Statistical data for individual experiments are presented in the appropriate figure legends. In all cases, ‘*n*’ is the number of independent experimental repeats from which the mean ± s.e.m. has been calculated.

## Data accessibility

All data supporting our research are included in the submission.

## References

[1] Jares P, Blow JJ. 2000 *Xenopus* cdc7 function is dependent on licensing but not on XORC, XCdc6, or CDK activity and is required for XCdc45 loading. Genes Dev. **14**, 1528-1540. 10859170 PMC316685

[2] Walter JC. 2000 Evidence for sequential action of cdc7 and cdk2 protein kinases during initiation of DNA replication in *Xenopus* egg extracts. J. Biol. Chem. **275**, 39 773-39 778. (10.1074/jbc.M008107200) 11005825

[3] Poh WT, Chadha GS, Gillespie PJ, Kaldis P, Blow JJ. 2014 *Xenopus* Cdc7 executes its essential function early in S phase and is counteracted by checkpoint-regulated protein phosphatase 1. Open Biol. **4**, 130138. (10.1098/rsob.130138) 24403013 PMC3909274

[4] Alver RC, Chadha GS, Gillespie PJ, Blow JJ. 2017 Reversal of DDK-mediated MCM phosphorylation by Rif1-PP1 regulates replication initiation and replisome stability independently of ATR/Chk1. Cell Rep. **18**, 2508-2520. (10.1016/j.celrep.2017.02.042) 28273463 PMC5357733

[5] Muramatsu S, Hirai K, Tak YS, Kamimura Y, Araki H. 2010 CDK-dependent complex formation between replication proteins Dpb11, Sld2, Pol (epsilon), and GINS in budding yeast. Genes Dev. **24**, 602-612. (10.1101/gad.1883410) 20231317 PMC2841337

[6] Li Y, Araki H. 2013 Loading and activation of DNA replicative helicases: the key step of initiation of DNA replication. Genes Cells **18**, 266-277. (10.1111/gtc.12040) 23461534 PMC3657122

[7] Yeeles JT, Deegan TD, Janska A, Early A, Diffley JF. 2015 Regulated eukaryotic DNA replication origin firing with purified proteins. Nature **519**, 431-435. (10.1038/nature14285) 25739503 PMC4874468

[8] Deegan TD, Yeeles JT, Diffley JF. 2016 Phosphopeptide binding by Sld3 links Dbf4-dependent kinase to MCM replicative helicase activation. EMBO J. **35**, 961-973. (10.15252/embj.201593552) 26912723 PMC4864760

[9] Hiraga S et al. 2014 Rif1 controls DNA replication by directing Protein Phosphatase 1 to reverse Cdc7-mediated phosphorylation of the MCM complex. Genes Dev. **28**, 372-383. (10.1101/gad.231258.113) 24532715 PMC3937515

[10] Hiraga SI et al. 2017 Human RIF1 and protein phosphatase 1 stimulate DNA replication origin licensing but suppress origin activation. EMBO Rep. **18**, 403-419. (10.15252/embr.201641983) 28077461 PMC5331243

[11] Kamimura Y, Masumoto H, Sugino A, Araki H. 1998 Sld2, which interacts with Dpb11 in *Saccharomyces cerevisiae*, is required for chromosomal DNA replication. Mol Cell Biol. **18**, 6102-6109. (10.1128/mcb.18.10.6102) 9742127 PMC109196

[12] Tanaka S, Nakato R, Katou Y, Shirahige K, Araki H. 2011 Origin association of Sld3, Sld7, and Cdc45 proteins is a key step for determination of origin-firing timing. Curr. Biol. **21**, 2055-2063. (10.1016/j.cub.2011.11.038) 22169533

[13] Tanaka T, Umemori T, Endo S, Muramatsu S, Kanemaki M, Kamimura Y, Obuse C, Araki H. 2011b Sld7, an Sld3-associated protein required for efficient chromosomal DNA replication in budding yeast. EMBO J. **30**, 2019-2030. (10.1038/emboj.2011.115) 21487389 PMC3098486

[14] Itou H, Shirakihara Y, Araki H. 2015 The quaternary structure of the eukaryotic DNA replication proteins Sld7 and Sld3. Acta Crystallogr. D Biol. Crystallogr. **71**, 1649-1656. (10.1107/S1399004715010457) 26249346

[15] Boyd MT, Vlatkovic N, Haines DS. 2000 A novel cellular protein (MTBP) binds to MDM2 and induces a G1 arrest that is suppressed by MDM2. J. Biol. Chem. **275**, 31 883-31 890. (10.1074/jbc.M004252200) 10906133

[16] Odvody J et al. 2010 A deficiency in Mdm2 binding protein inhibits Myc-induced B-cell proliferation and lymphomagenesis. Oncogene **29**, 3287-3296. (10.1038/onc.2010.82) 20305689 PMC2880662

[17] Grieb BC, Gramling MW, Arrate MP, Chen X, Beauparlant SL, Haines DS, Xiao H, Eischen CM. 2014 Oncogenic protein MTBP interacts with MYC to promote tumorigenesis. Cancer Res. **74**, 3591-3602. (10.1158/0008-5472.CAN-13-2149) 24786788 PMC4079748

[18] Grieb BC, Chen X, Eischen CM. 2014 MTBP is overexpressed in triple-negative breast cancer and contributes to its growth and survival. Mol. Cancer Res. **12**, 1216-1224. (10.1158/1541-7786.MCR-14-0069) 24866769 PMC4163510

[19] Boos D, Yekezare M, Diffley JF. 2013 Identification of a heteromeric complex that promotes DNA replication origin firing in human cells. Science **340**, 981-984. (10.1126/science.1237448) 23704573

[20] Kumagai A, Shevchenko A, Shevchenko A, Dunphy WG. 2011 Direct regulation of Treslin by cyclin-dependent kinase is essential for the onset of DNA replication. J. Cell Biol. **193**, 995-1007. (10.1083/jcb.201102003) 21646402 PMC3115804

[21] Sansam CG, Goins D, Siefert JC, Clowdus EA, Sansam CL. 2015 Cyclin-dependent kinase regulates the length of S phase through TICRR/TRESLIN phosphorylation. Genes Dev **29**, 555-566. (10.1101/gad.246827.114) 25737283 PMC4358407

[22] Im JS, Park SY, Cho WH, Bae SH, Hurwitz J, Lee JK. 2015 RecQL4 is required for the association of Mcm10 and Ctf4 with replication origins in human cells. Cell Cycle **14**, 1001-1009. (10.1080/15384101.2015.1007001) 25602958 PMC4612657

[23] Kumagai A, Dunphy WG. 2017 MTBP, the partner of Treslin, contains a novel DNA-binding domain that is essential for proper initiation of DNA replication. Mol. Biol. Cell **28**, 2998-3012. (10.1091/mbc.E17-07-0448) 28877985 PMC5662258

[24] Gillespie PJ, Neusiedler J, Creavin K, Chadha GS, Blow JJ. 2016 Cell cycle synchronization in *Xenopus* egg extracts. Methods Mol. Biol **1342**, 101-147. (10.1007/978-1-4939-2957-3_6) 26254920

[25] Siegel LM, Monty KJ. 1966 Determination of molecular weights and frictional ratios of proteins in impure systems by use of gel filtration and density gradient centrifugation: application to crude preparations of sulfite and hydroxylamine reductases. Biochim. Biophys. Acta **112**, 346-362. (10.1016/0926-6585(66)90333-5) 5329026

[26] Blow JJ, Laskey RA. 1986 Initiation of DNA replication in nuclei and purified DNA by a cell-free extract of *Xenopus* eggs. Cell **47**, 577-587. (10.1016/0092-8674(86)90622-7) 3779837

[27] Blow JJ, Gillespie PJ, Francis D, Jackson DA. 2001 Replication origins in *Xenopus* egg extract are 5–15 kilobases apart and are activated in clusters that fire at different times. J. Cell Biol. **152**, 15-25. (10.1083/jcb.152.1.15) 11149917 PMC2193667

[28] McGarry TJ, Kirschner MW. 1998 Geminin, an inhibitor of DNA replication, is degraded during mitosis. Cell **93**, 1043-1053. (10.1016/s0092-8674(00)81209-x) 9635433

[29] Tada S, Li A, Maiorano D, Mechali M, Blow JJ. 2001 Repression of origin assembly in metaphase depends on inhibition of RLF-B/Cdt1 by geminin. Nat. Cell Biol. **3**, 107-113. (10.1038/35055000) 11175741 PMC3605706

[30] Wohlschlegel JA, Dwyer BT, Dhar SK, Cvetic C, Walter JC, Dutta A. 2000 Inhibition of eukaryotic DNA replication by geminin binding to Cdt1. Science **290**, 2309-2312. (10.1126/science.290.5500.2309) 11125146

[31] Gardner NJ, Gillespie PJ, Carrington JT, Shanks EJ, McElroy SP, Haagensen EJ, Frearson JA, Woodland A, Blow JJ. 2017 The high-affinity interaction between ORC and DNA that is required for replication licensing is inhibited by 2-arylquinolin-4-amines. Cell Chem. Biol. **24**, 981-992. (10.1016/j.chembiol.2017.06.019) 28781123 PMC5563080

[32] Blow JJ, Nurse P. 1990 A cdc2-like protein is involved in the initiation of DNA replication in *Xenopus* egg extracts. Cell **62**, 855-862. (10.1016/0092-8674(90)90261-c) 2203536

[33] Fang F, Newport JW. 1991 Evidence that the G1-S and G2-M transitions are controlled by different cdc2 proteins in higher eukaryotes. Cell **66**, 731-742. (10.1016/0092-8674(91)90117-h) 1652371

[34] Strausfeld UP, Howell M, Rempel R, Maller JL, Hunt T, Blow JJ. 1994 Cip1 blocks the initiation of DNA replication in *Xenopus* extracts by inhibition of cyclin-dependent kinases. Curr. Biol. **4**, 876-883. (10.1016/s0960-9822(00)00196-2) 7850420

[35] Masumoto H, Muramatsu S, Kamimura Y, Araki H. 2002 S-Cdk-dependent phosphorylation of Sld2 essential for chromosomal DNA replication in budding yeast. Nature **415**, 651-655. (10.1038/nature713) 11807498

[36] Tanaka S, Umemori T, Hirai K, Muramatsu S, Kamimura Y, Araki H. 2007 CDK-dependent phosphorylation of Sld2 and Sld3 initiates DNA replication in budding yeast. Nature **445**, 328-332. (10.1038/nature05465) 17167415

[37] Zegerman P, Diffley JF. 2007 Phosphorylation of Sld2 and Sld3 by cyclin-dependent kinases promotes DNA replication in budding yeast. Nature **445**, 281-285. (10.1038/nature05432) 17167417

[38] Kumagai A, Shevchenko A, Shevchenko A, Dunphy WG. 2010 Treslin collaborates with TopBP1 in triggering the initiation of DNA replication. Cell **140**, 349-359. (10.1016/j.cell.2009.12.049) 20116089 PMC2857569

[39] Natsume T et al. 2013 Kinetochores coordinate pericentromeric cohesion and early DNA replication by Cdc7-Dbf4 kinase recruitment. Mol. Cell **50**, 661-674. (10.1016/j.molcel.2013.05.011) 23746350 PMC3679449

[40] Montagnoli A et al. 2008 A Cdc7 kinase inhibitor restricts initiation of DNA replication and has antitumor activity. Nat. Chem. Biol. **4**, 357-365. (10.1038/nchembio.90) 18469809

[41] Finlay DR, Newmeyer DD, Price TM, Forbes DJ. 1987 Inhibition of in vitro nuclear transport by a lectin that binds to nuclear pores. J. Cell Biol. **104**, 189-200. (10.1083/jcb.104.2.189) 3805121 PMC2114419

[42] Rowles A, Tada S, Blow JJ. 1999 Changes in association of the *Xenopus* origin recognition complex with chromatin on licensing of replication origins. J. Cell Sci. **112**, 2011-2018. (10.1242/jcs.112.12.2011) 10341218 PMC3605702

[43] Iwai K et al. 2019 Molecular mechanism and potential target indication of TAK-931, a novel CDC7-selective inhibitor. Sci. Adv. **5**, eaav3660. (10.1126/sciadv.aav3660) 31131319 PMC6531005

[44] Sheu YJ, Stillman B. 2006 Cdc7-Dbf4 phosphorylates MCM proteins via a docking site-mediated mechanism to promote S phase progression. Mol. Cell **24**, 101-113. (10.1016/j.molcel.2006.07.033) 17018296 PMC2923825

[45] Boos D, Sanchez-Pulido L, Rappas M, Pearl LH, Oliver AW, Ponting CP, Diffley JF. 2011 Regulation of DNA replication through Sld3-Dpb11 interaction is conserved from yeast to humans. Curr. Biol. **21**, 1152-1157. (10.1016/j.cub.2011.05.057) 21700459

[46] Mantiero D, Mackenzie A, Donaldson A, Zegerman P. 2011 Limiting replication initiation factors execute the temporal programme of origin firing in budding yeast. EMBO J. **30**, 4805-4814. (10.1038/emboj.2011.404) 22081107 PMC3243606

[47] Collart C, Allen GE, Bradshaw CR, Smith JC, Zegerman P. 2013 Titration of four replication factors is essential for the *Xenopus laevis* midblastula transition. Science **341**, 893-896. (10.1126/science.1241530) 23907533 PMC3898016

[48] Gambus A, Jones RC, Sanchez-Diaz A, Kanemaki M, van Deursen F, Edmondson RD, Labib K. 2006 GINS maintains association of Cdc45 with MCM in replisome progression complexes at eukaryotic DNA replication forks. Nat. Cell Biol. **8**, 358-366. (10.1038/ncb1382) 16531994

[49] Kanemaki M, Labib K. 2006 Distinct roles for Sld3 and GINS during establishment and progression of eukaryotic DNA replication forks. EMBO J. **25**, 1753-1763. (10.1038/sj.emboj.7601063) 16601689 PMC1440835

[50] Luciani MG, Oehlmann M, Blow JJ. 2004 Characterization of a novel ATR-dependent, Chk1-independent, intra-S-phase checkpoint that suppresses initiation of replication in *Xenopus*. J. Cell Sci. **117**, 6019-6030. (10.1242/jcs.01400) 15536124 PMC2701543

[51] Woodward AM, Gohler T, Luciani MG, Oehlmann M, Ge X, Gartner A, Jackson DA, Blow JJ. 2006 Excess Mcm2-7 license dormant origins of replication that can be used under conditions of replicative stress. J. Cell Biol. **173**, 673-683. (10.1083/jcb.200602108) 16754955 PMC2063885

[52] Ge XQ, Jackson DA, Blow JJ. 2007 Dormant origins licensed by excess Mcm2-7 are required for human cells to survive replicative stress. Genes Dev. **21**, 3331-3341. (10.1101/gad.457807) 18079179 PMC2113033

[53] Ibarra A, Schwob E, Mendez J. 2008 Excess MCM proteins protect human cells from replicative stress by licensing backup origins of replication. Proc. Natl Acad. Sci. USA **105**, 8956-8961. (10.1073/pnas.0803978105) 18579778 PMC2449346

[54] Blow JJ, Ge XQ. 2009 A model for DNA replication showing how dormant origins safeguard against replication fork failure. EMBO Rep. **10**, 406-412. (10.1038/embor.2009.5) 19218919 PMC2644062

[55] Ge XQ, Blow JJ. 2010 Chk1 inhibits replication factory activation but allows dormant origin firing in existing factories. J. Cell Biol. **191**, 1285-1297. (10.1083/jcb.201007074) 21173116 PMC3010067

[56] Mahbubani HM, Chong JP, Chevalier S, Thommes P, Blow JJ. 1997 Cell cycle regulation of the replication licensing system: involvement of a Cdk-dependent inhibitor. J. Cell Biol. **136**, 125-135. (10.1083/jcb.136.1.125) 9008708 PMC2132454

[57] Edwards MC, Tutter AV, Cvetic C, Gilbert CH, Prokhorova TA, Walter JC. 2002 MCM2-7 complexes bind chromatin in a distributed pattern surrounding the origin recognition complex in *Xenopus* egg extracts. J. Biol. Chem. **277**, 33 049-33 057. (10.1074/jbc.M204438200) 12087101

[58] Gambus A, Khoudoli GA, Jones RC, Blow JJ. 2011 MCM2-7 form double hexamers at licensed origins in *Xenopus* egg extract. J. Biol. Chem. **286**, 11 855-11 864. (10.1074/jbc.M110.199521) PMC3064236 21282109

[59] Prokhorova TA, Blow JJ. 2000 Sequential MCM/P1 subcomplex assembly is required to form a heterohexamer with replication licensing activity. J. Biol. Chem. **275**, 2491-2498. (10.1074/jbc.275.4.2491) 10644704 PMC3626232

[60] Rowles A, Chong JP, Brown L, Howell M, Evan GI, Blow JJ. 1996 Interaction between the origin recognition complex and the replication licensing system in *Xenopus*. Cell **87**, 287-296. (10.1016/s0092-8674(00)81346-x) 8861912

[61] Oehlmann M, Score AJ, Blow JJ. 2004 The role of Cdc6 in ensuring complete genome licensing and S phase checkpoint activation. J. Cell Biol. **165**, 181-190. (10.1083/jcb.200311044) 15096526 PMC2172031

[62] Yan H, McCane J, Toczylowski T, Chen C. 2005 Analysis of the *Xenopus* Werner syndrome protein in DNA double-strand break repair. J. Cell Biol. **171**, 217-227. (10.1083/jcb.200502077) 16247024 PMC2171202

[63] Thomson AM, Gillespie PJ, Blow JJ. 2010 Replication factory activation can be decoupled from the replication timing program by modulating Cdk levels. J. Cell Biol. **188**, 209-221. (10.1083/jcb.200911037) 20083602 PMC2812520

[64] Gillespie PJ, Gambus A, Blow JJ. 2012 Preparation and use of *Xenopus* egg extracts to study DNA replication and chromatin associated proteins. Methods **57**, 203-213. (10.1016/j.ymeth.2012.03.029) 22521908 PMC3437562

[65] Chong JP, Thommes P, Rowles A, Mahbubani HM, Blow JJ. 1997 Characterization of the *Xenopus* replication licensing system. Methods Enzymol. **283**, 549-564. (10.1016/s0076-6879(97)83043-1) 9251047

[66] Ferenbach A, Li A, Brito-Martins M, Blow JJ. 2005 Functional domains of the *Xenopus* replication licensing factor Cdt1. Nucleic Acids Res. **33**, 316-324. (10.1093/nar/gki176) 15653632 PMC546161

